# Disturbed neurotransmitter homeostasis in ether lipid deficiency

**DOI:** 10.1093/hmg/ddz040

**Published:** 2019-02-13

**Authors:** Fabian Dorninger, Theresa König, Petra Scholze, Michael L Berger, Gerhard Zeitler, Christoph Wiesinger, Anna Gundacker, Daniela D Pollak, Sigismund Huck, Wilhelm W Just, Sonja Forss-Petter, Christian Pifl, Johannes Berger

**Affiliations:** 1 Department of Pathobiology of the Nervous System, Center for Brain Research, Medical University of Vienna, Spitalgasse 4, Vienna, Austria; 2 Department of Molecular Neurosciences, Center for Brain Research, Medical University of Vienna, Spitalgasse 4, Vienna, Austria; 3 Department of Neurophysiology and Neuropharmacology, Center for Physiology and Pharmacology, Medical University of Vienna, Schwarzspanierstraße 17, Vienna, Austria; 4 Biochemistry Center Heidelberg (BZH), University of Heidelberg, Im Neuenheimer Feld 328, Heidelberg, Germany

## Abstract

Plasmalogens, the most prominent ether (phospho)lipids in mammals, are structural components of most cellular membranes. Due to their physicochemical properties and abundance in the central nervous system, a role of plasmalogens in neurotransmission has been proposed, but conclusive data are lacking. Here, we targeted this issue in the glyceronephosphate O-acyltransferase (*Gnpat*) KO mouse, a model of complete deficiency in ether lipid biosynthesis. Throughout the study, focusing on adult male animals, we found reduced brain levels of various neurotransmitters. In the dopaminergic nigrostriatal tract, synaptic endings but not neuronal cell bodies were affected. Neurotransmitter turnover was altered in ether lipid-deficient murine as well as human *post-mortem* brain tissue. A generalized loss of synapses did not account for the neurotransmitter deficits, since the levels of several presynaptic proteins appeared unchanged. However, reduced amounts of vesicular monoamine transporter indicate a compromised vesicular uptake of neurotransmitters. As exemplified by norepinephrine, the release of neurotransmitters from *Gnpat* KO brain slices was diminished in response to strong electrical and chemical stimuli. Finally, addressing potential phenotypic correlates of the disturbed neurotransmitter homeostasis, we show that ether lipid deficiency manifests as hyperactivity and impaired social interaction. We propose that the lack of ether lipids alters the properties of synaptic vesicles leading to reduced amounts and release of neurotransmitters. These features likely contribute to the behavioral phenotype of *Gnpat* KO mice, potentially modeling some human neurodevelopmental disorders like autism or attention deficit hyperactivity disorder.

## Introduction

In chemical neurotransmission, the presynaptic release of neurotransmitters is controlled by the trafficking of synaptic vesicles in a complex multi-step vesicle cycle. Most studies have focused on the major proteins in this procedure, but also lipids play a crucial role in the regulation of the synaptic vesicle cycle (1), both as constituents of the involved membrane compartments and as source of signaling mediators. In molecular terms, the large variety of membrane lipid species allows for fine-tuning of biophysical membrane properties to the requirements of different subcellular compartments or molecular events (2).

In the synaptic vesicle cycle, uptake of neurotransmitters into vesicles is accomplished by transmitter-specific transporters, which operate using an electrochemical proton (H^+^) gradient generated by a V-type ATPase (3). Synaptic vesicle exocytosis can be subdivided into vesicle docking, priming and fusion. The soluble N-ethylmaleimide–sensitive factor attachment protein receptor (SNARE) complex consisting of both vesicular proteins and plasma membrane proteins plays a prominent role in these processes. With the support of a large number of auxiliary proteins and factors, three of the SNARE proteins, synaptobrevin (at the synaptic vesicle membrane), syntaxin and SNAP-25 (at the presynaptic membrane) mediate exocytosis (4). Synaptotagmin acts as a sensor of Ca^2+^ (5), which enters the cell through voltage-gated Ca^2+^ channels in response to an incoming action potential, and relays the Ca^2+^ signal to the SNARE complex, enabling membrane fusion and transmitter release. Most of the proteins involved in synaptic release are membrane-associated and preferred membrane lipids provide their correct functional environment. For example, small membrane domains termed lipid rafts have been suggested to accomplish sorting and sequestration of SNARE proteins (6) and to accommodate postsynaptic receptors (7).

After exocytosis, the synaptic vesicles are recycled and refilled with neurotransmitters via either a clathrin-dependent endosomal pathway, the clathrin-independent ‘kiss-and-run’ mechanism, or, possibly, while staying docked to the membrane (‘kiss-and-stay’) (8). Following release, neurotransmitters are rapidly cleared from the synaptic cleft by either enzymatic degradation or by cellular uptake. The latter is accomplished by transporters located in the membranes of presynaptic (in some cases also postsynaptic) neurons or of surrounding glial cells to act as important regulators of the neurotransmission process (9). The complex trafficking steps of the whole cycle are tightly controlled, and the dynamics of the endo- and exocytotic events crucially depends on the detailed molecular composition of membranes (10), emphasizing the importance of lipid homeostasis in this process.

In the present study, we examine the impact of a specific class of synaptic lipids, the ether (phospho)lipids, which, due to their unique biophysical properties, are of particular importance for the synaptic vesicle cycle (11). These glycerophospholipids have an ether bond at the *sn*-1 position of the glycerol backbone, in contrast to the ester bond in the more common diacyl phospholipids. The biosynthesis of ether lipids is initiated in peroxisomes, while later steps require the concerted action of various organelles (12). Plasmalogens, the most abundant subtype of ether lipids, are present in comparatively large amounts in the central nervous system (CNS) (11) and are a major component of both synaptic membranes (13) and synaptic vesicles (14). They are enriched in lipid rafts (15) and have been implicated in membrane fusion and constriction processes (16,17). Moreover, the high prevalence of polyunsaturated fatty acids (PUFAs) in plasmalogens may be of particular significance, as the presence of PUFAs increases the fluidity and flexibility of synaptic membranes (10). In addition, these fatty acids are essential for proper development of the brain and cognition (18). Increasing evidence implicates also platelet-activating factor, another ether lipid, as an important regulator of synaptic processes [reviewed in (11)].

Researchers have long been speculating about a role of ether lipids in the synaptic vesicle cycle and in neurotransmission (19–22), but their impact on these processes *in vivo* remains largely unclear. Here, we utilize the ether lipid-deficient glyceronephosphate O-acyltransferase (*Gnpat*) KO mouse as tool to study the role of ether lipids in neurotransmitter homeostasis and neurotransmission *in vivo*. Ether lipid deficiency in mice manifests in growth retardation, ocular deficits (particularly cataracts) and male infertility (23); impaired peripheral myelination (24) and alterations in cerebellar structure (25). Recently, our own work revealed deficits in motor performance and muscle strength, accompanied by structural and functional changes at the neuromuscular junction of *Gnpat* KO mice (26). A previous study using synaptosomes derived from this mouse model already yielded valuable insight into properties of synaptic release under conditions of ether lipid deficiency (25). We now extend these findings and demonstrate that lack of ether lipids leads to considerable consequences for the synaptic vesicle cycle with a more generalized reduction of neurotransmitters and aberrant neurotransmitter release. These observations, underpinned by a hyperactive phenotype with deficient social interaction in ether lipid-deficient mice, provide a potential mechanistic link between ether lipid deficiency and neurodevelopmental disorders associated with hyperactive and stereotypic behavior.

## Results

### Gnpat KO mice have brain deficits in a wide range of neurotransmitters

**Figure 1:**
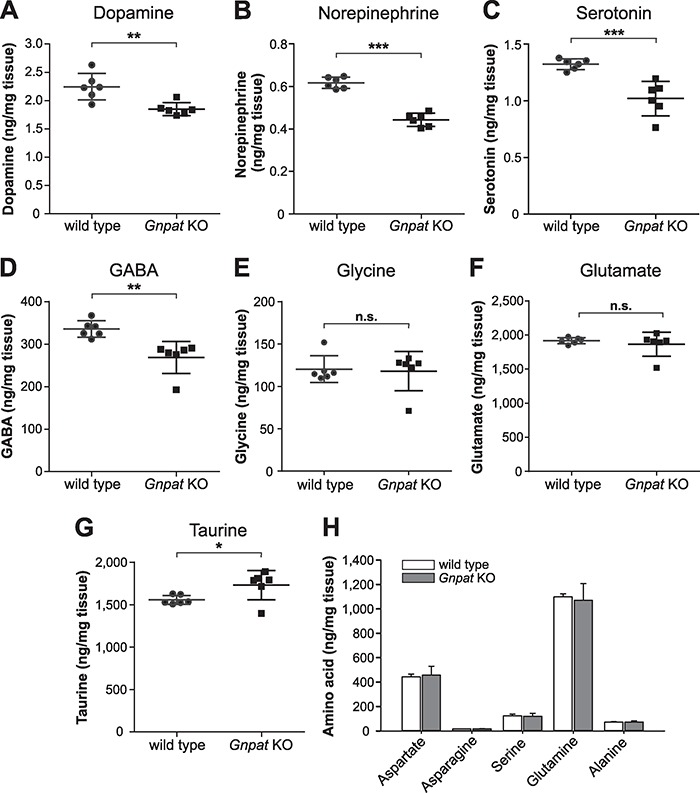
Ether lipid deficiency manifests as neurotransmitter deficits in the murine cerebrum. The levels of dopamine (**A**), norepinephrine (**B**), serotonin (**C**), GABA (**D**), glycine (**E**), glutamate (**F**) and taurine (**G**) were determined in cerebral homogenates from WT and *Gnpat* KO mice (A–H, *n* = 6 for both genotypes) by using HPLC. Statistical analysis was performed using two-tailed Student’s *t*-tests. Graphs depict individual data together with group mean ± SD. (**H**) For control reasons, the levels of several proteinogenic amino acids were analyzed. Statistical analysis using two-tailed Student’s *t*-tests did not reveal any significant differences (no correction for multiple comparisons due to the absence of significant results). Bars represent group mean ± SD. ^***^P < 0.001; ^**^P < 0.01; ^*^P < 0.05; *n.s.*, not significant. The displayed dopamine and serotonin data were used for the calculation of the neurotransmitter-to-metabolite ratios presented in Table 1.

In an initial screen, we prepared homogenates from the cerebrum of wild-type (WT) and *Gnpat* KO mice and analyzed the tissue levels of the classical monoamine neurotransmitters dopamine, norepinephrine and serotonin as well as the amino acid neurotransmitters GABA, glycine, glutamate and taurine by high-performance liquid chromatography (HPLC). Of the monoamines, dopamine exhibited the highest mean tissue concentration; however, we found robustly decreased mean dopamine levels (−24%) in the brains of *Gnpat* KO mice compared to WT mice (Fig. 1A). Strikingly, we obtained similar results for the other two monoamine neurotransmitters analyzed, norepinephrine (−28%, Fig. 1B) and serotonin (−23%, Fig. 1C). Furthermore, we detected a reduction by 20% of GABA, the major inhibitory neurotransmitter in the CNS, in *Gnpat* KO mice (Fig. 1D), close to the decrease in monoamine neurotransmitters. We could not detect any statistically significant difference between the genotypes in the amounts of glycine (Fig. 1E) and glutamate (Fig. 1F), two neurotransmitters also serving as proteinogenic amino acids. Notably, we found significantly increased levels of the unconventional neurotransmitter and neuromodulatory amino sulfonic acid taurine (+12%), the second most abundant endogenous amino acid in the brain after glutamate, in cerebral homogenates from *Gnpat* KO mice compared to WT controls (Fig. 1G). However, because taurine differs from classical neurotransmitters in several respects—e.g. it is released from the cell soma, not from synaptic vesicles (27)—the taurine levels should be viewed independently from the changes in other neurotransmitters. Due to the wide range of neuroprotective functions of taurine (28), the increased levels in the brain of *Gnpat* KO mice may derive from attempts to shield neurons from damage otherwise resulting from ether lipid deficiency.

*Gnpat* KO mice generally have a smaller body size, including brain mass [mean brain weight ± standard deviation (SD): WT, 478 ± 29 mg; *Gnpat* KO, 383 ± 24 mg; *n* = 18 per genotype; *P* < 0.0001 (two-tailed Student’s *t*-test)]. Thus, to rule out general effects due to size differences, we also measured the levels of several proteinogenic amino acids without major neurotransmitter function. The levels of these compounds were equal between the two genotypes (Fig. 1H).

In order to gain further insights into the nature of the neurotransmitter reduction in ether lipid-deficient animals, we next analyzed their distribution in three distinct brain regions: the parietal cortex, the substantia nigra and the (corpus) striatum. Because GABAergic synapses are abundant in all three regions, by measuring GABA levels, we should be able to assess whether neurotransmitter loss in ether lipid-deficient mice is a ubiquitous phenomenon or restricted to specific brain areas. Consistent with our findings in cerebral homogenates, the GABA levels were clearly reduced in the parietal cortex by more than 20% in *Gnpat* KO mice compared to WT mice and to a similar extent in the substantia nigra but not in striatum (Supplementary Material, Fig. S1A). Strikingly, the tissue concentration of taurine was consistently increased by 15–20% in all the three brain regions (Supplementary Material, Fig. S1B), although it did not quite reach statistical significance in the striatum (*P* = 0.055). By analogy with the analyses of whole cerebrum, we also measured the levels of several proteinogenic amino acids in these three brain areas as a control but did not find any differences between the two genotypes (data not shown).

For norepinephrine and serotonin, the depletion was even more pronounced in the *Gnpat* KO parietal cortex than in the whole cerebral homogenates (Supplementary Material, Fig. S1C and D), with 37% and 46% lower norepinephrine and serotonin levels, respectively, in homogenates from *Gnpat* KO mice as compared to WT mice. Decreased mean levels were also seen in the other brain regions, except for norepinephrine in the striatum, but this trend did not reach statistical significance (Supplementary Material, Fig. S1C and D). Together, these results demonstrate generally lowered neurotransmitter levels in several brain regions of *Gnpat* KO mice.

**Figure 2:**
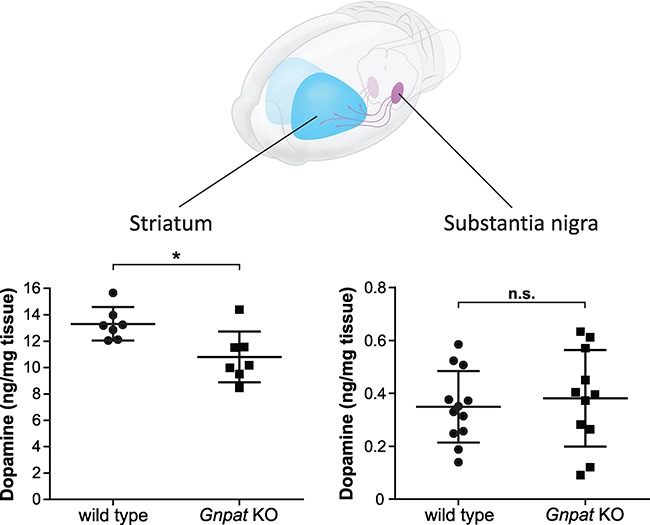
A synapse-related problem accounts for the dopamine deficit in the ether lipid-deficient mouse brain. The cartoon at the top illustrates the nigrostriatal pathway with cell bodies of dopaminergic neurons in the substantia nigra and their axon terminals in the striatum. Dopamine levels were determined in the striatum (left panel; *n* = 7/genotype) and the substantia nigra (right panel; WT: *n* = 12, *Gnpat* KO: *n* = 11) of WT and *Gnpat* KO mice by using HPLC. Graphs depict individual data together with group mean ± SD. Statistical analysis was performed using two-tailed Student’s *t*-tests. ^*^P < 0.05; *n.s.*, not significant. The displayed data were used for the calculation of the neurotransmitter-to-metabolite ratios presented in Table 1.

Next, we raised the question whether the loss of neurotransmitters is specific for axon terminals or also affects their biosynthesis in the neuronal cell bodies. For this, we focused on the dopaminergic nigrostriatal pathway, in which dopaminergic cell bodies are abundantly located in the substantia nigra while their axons project to the striatum, where a large number of dopaminergic synapses is found (Fig. 2). Consequently, in the substantia nigra, a considerable amount of dopamine is present in the cytosol (29), whereas in the striatum, dopamine is highly enriched in synaptic vesicles. Remarkably, we found a 19% depletion of dopamine in the striatum of *Gnpat* KO mice compared to WT levels (Fig. 2, left panel), similar to that observed in the whole cerebrum. In contrast, dopamine levels in the substantia nigra did not differ between WT and ether lipid-deficient animals (Fig. 2, right panel), indicating that the depletion of dopamine and probably of the other neurotransmitters as well is restricted to the synapse.

### A general loss of synapses does not account for the reduced neurotransmitter levels

**Figure 3:**
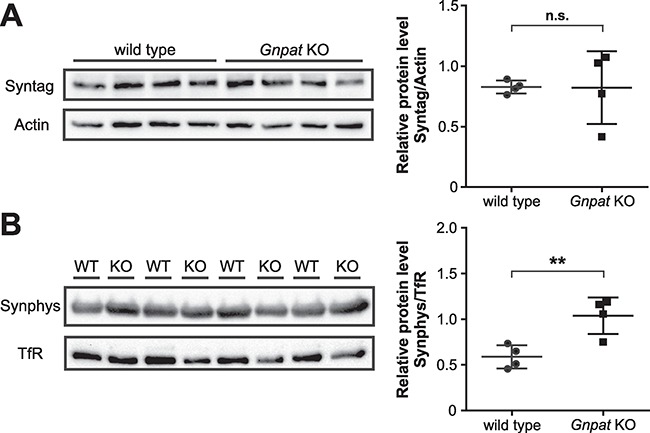
No general synapse loss in ether lipid-deficient mouse brains. (**A**) Western blot analysis of cortical membrane–enriched extracts (5 μg per lane) derived from WT and *Gnpat* KO mice (*n* = 4/genotype) testing for the amounts of synaptotagmin I (Syntag, A) and synaptophysin (Synphys, **B**). Immunoblots were stripped and reprobed with actin (A) and the membrane marker transferrin receptor (TfR, B) as loading controls. Densitometric quantification is shown as individual data together with group mean ± SD. Statistical analysis was performed using two-tailed Student’s *t*-tests. ^**^P < 0.01; *n.s.*, not significant. Full blots for the presented data can be found in Figure S2.

Two independent groups have previously shown a minor loss of axons, particularly in the cerebellum, of *Gnpat* KO mice (30), and a marked reduction of axons in the peripheral nervous system of 1.5-year-old ether lipid-deficient mice (24). In order to assess potential loss of synapses in the CNS of adult ether lipid-deficient mice, we applied western blot analysis for relative quantification of synaptic marker proteins in the brain. We found no alterations between the two genotypes in the levels of synaptotagmin I, a presynaptic protein that relays the Ca^2+^ signal of an incoming action potential to the SNARE complex, in membrane fractions derived from cerebral cortex homogenates (Fig. 3A and Supplementary Material, Fig. S2A). Also, the amount of synaptophysin, a protein of the SNARE complex that is localized to synaptic vesicles, was inconsistent with loss of synapses in the cortex of *Gnpat* KO mice. In contrast, when normalized to the ubiquitous transmembrane protein transferrin receptor, the densitometric quantification of synaptophysin bands rather showed an increase in *Gnpat* KO extracts (Fig. 3B and Supplementary Material, Fig. S2B). These results argue against a general loss of synapses in the brain of adult *Gnpat* KO mice.

### Ether lipid deficiency causes increased turnover of monoamine neurotransmitters

Next, we quantified the steady-state levels of the main metabolites of monoamine neurotransmitters in the whole cerebral homogenates and the dissected brain regions by HPLC. As described previously (31), for each sample, we determined the metabolite/transmitter ratio to calculate the fraction of metabolized transmitter molecules as an estimate of monoamine neurotransmitter turnover. The levels of 5-hydroxyindoleacetic acid (5-HIAA), the main degradation product of serotonin, did not differ between WT and *Gnpat* KO mice in whole cerebrum or parietal cortex (Table 1). However, the ratio of 5-HIAA to serotonin, as a measure of serotonin turnover, was significantly increased in total cerebral as well as parietal cortex homogenates of *Gnpat* KO mice. For dopamine turnover, we obtained a similar pattern: the levels of the main dopamine metabolites 3,4-dihydroxyphenylacetic acid (DOPAC) and homovanillic acid (HVA) were largely unchanged in both total cerebrum and striatum of WT and *Gnpat* KO mice (Table 1). However, due to the markedly decreased dopamine levels in *Gnpat* KO mice, the metabolite/dopamine ratio was increased for both metabolites and brain regions (except for HVA/DA in the striatum). Altogether, we uncovered increased metabolite/monoamine neurotransmitter ratios in the brain of ether lipid-deficient mice, which apparently is a consequence of the normal metabolite levels, but significantly reduced neurotransmitter levels.

**Table 1:** Levels of monoamine metabolites and metabolite-to-neurotransmitter ratios in whole cerebrum and selected brain regions of WT and *Gnpat* KO mice

**Metabolite (brain region)**	**WT**	***Gnpat* KO**
*Serotonin metabolites*		
5-HIAA (cerebrum^a^) (ng/mg tissue)	0.388 ± 0.043	0.397 ± 0.067
5-HIAA/5-HT^d^ (cerebrum^a^) (molar ratio)	0.270 ± 0.033	0.359 ± 0.032^***^
5-HIAA (parietal cortex^b^) (ng/mg tissue)	0.137 ± 0.032	0.113 ± 0.024
5-HIAA/5-HT^d^ (parietal cortex^b^) (molar ratio)	0.210 ± 0.040	0.330 ± 0.119^**^
*Dopamine metabolites*		
DOPAC (cerebrum^a^) (ng/mg tissue)	0.123 ± 0.021	0.124 ± 0.013
DOPAC/DA^d^ (cerebrum^a^) (molar ratio)	0.067 ± 0.011	0.086 ± 0.004^**^
HVA (cerebrum^a^) (ng/mg tissue)	0.143 ± 0.017	0.139 ± 0.030
HVA/DA^d^ (cerebrum^a^) (molar ratio)	0.070 ± 0.009	0.088 ± 0.013^*^
DOPAC (striatum^c^) (ng/mg tissue)	1.158 ± 0.175	1.285 ± 0.307
DOPAC/DA^d^ (striatum^c^) (molar ratio)	0.079 ± 0.010	0.108 ± 0.019^**^
HVA (striatum^c^) (ng/mg tissue)	1.273 ± 0.280	1.056 ± 0.330
HVA/DA^d^ (striatum^c^) (molar ratio)	0.081 ± 0.019	0.082 ± 0.019
3-MT (striatum^c^) (ng/mg tissue)	0.487 ± 0.097	0.405 ± 0.083

Abbreviations: DA, dopamine; 5-HT, serotonin; 3-MT, 3-methoxytyramine

^a^ *n* = 6/genotype

^b^WT: *n* = 12, *Gnpat* KO: *n* = 11

^c^ *n* = 7/genotype

^d^Dopamine and serotonin values are derived from the data sets displayed in Figures 1 (cerebrum) and **2** (striatum). All values are mean ± SD. ^***^P < 0.001, ^**^P < 0.01, ^*^P < 0.05 (two-tailed Student’s *t*-test)

### Enhanced monoamine neurotransmitter turnover is partially replicated in human ether lipid-deficiency

To investigate neurotransmitter levels and turnover in human ether lipid deficiency, we obtained *post-mortem* brain tissue of patients with inborn ether lipid deficiency and controls from the NIH NeuroBioBank (University of Maryland, Baltimore, Maryland). By analogy to the mouse studies, we analyzed distinct brain regions: a defined region in the parietal cortex (Brodmann area 4) and the caudate nucleus. Since only one case of rhizomelic chondrodysplasia punctata (RCDP; representing isolated ether lipid deficiency) was available, we also included samples from nine cases of Zellweger spectrum disorder (a peroxisome biogenesis disorder, in which—next to ether lipid biosynthesis—also all other peroxisomal metabolic pathways are affected) and 24 age-matched controls in our study (for a description of the cases, see Supplementary Material, Table S1). The quantitative analysis of the neurotransmitters dopamine, norepinephrine, serotonin, GABA, glycine and glutamate in these tissue samples did not reveal any statistically significant differences between control and ether lipid-deficient samples in either the parietal cortex (Supplementary Material, Fig. S3) or the caudate nucleus (Supplementary Material, Fig. S4). This outcome was likely due to the heterogeneity of the cohorts and the strong variability of measurements in *post-mortem* samples. However, the control investigation of proteinogenic amino acids unveiled markedly increased glutamine levels in both brain regions of the patient group (Supplementary Material, Fig. S4H). As glutamine is enriched in astrocytes in the CNS, this finding may reflect the presence of astrocytosis, which has previously been observed in Zellweger spectrum patients (32).

Notably, like in the murine brain, we detected a trend toward increased turnover of monoamine neurotransmitters in the tissue of human ether lipid-deficient cases, particularly in the parietal cortex. In spite of considerable variability, compared to the control cases, we identified increased levels of the serotonin metabolite 5-HIAA and, consequently, an elevated ratio of 5-HIAA to serotonin in the patient samples. The levels of 5-HIAA were also significantly increased in the caudate nucleus of patients. Among the main dopamine metabolites in the parietal cortex, we found a statistically significant elevation of the DOPAC levels (Supplementary Material, Table S2). However, the analysis of dopamine metabolites in the caudate nucleus revealed no difference between control and ether lipid-deficient patient samples.

### Neurotransmitter release from ether lipid-deficient mouse brain slices is impaired upon high-intensity stimulation

**Figure 4:**
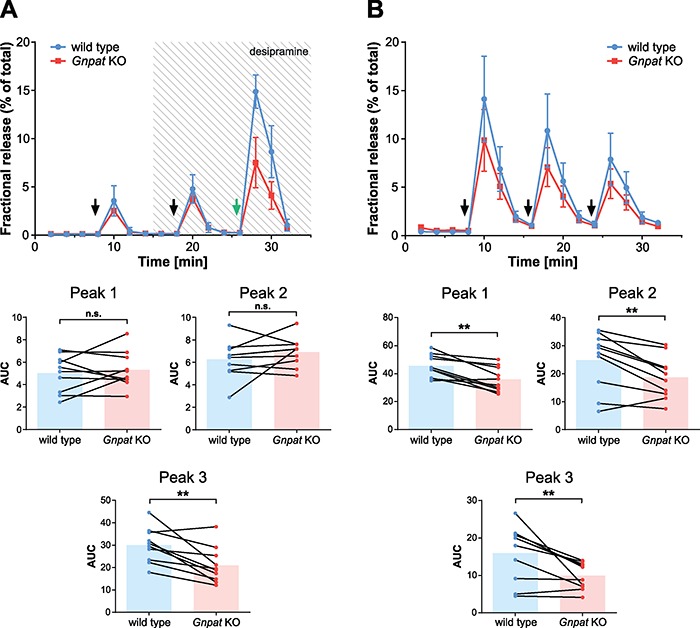
Norepinephrine release from brain slices is impaired upon ether lipid deficiency. (**A**) Hippocampal slices were loaded with [^3^H]-norepinephrine, and neurotransmitter release was induced by two consecutive electropulse series (100 pulses, 10 Hz; black arrows) and exposure to 40 mM KCl for 30 s (green arrow). The reuptake inhibitor desipramine was added after the first electrical stimulus (shaded area). The results of a representative experiment are shown in the upper panel. Each data point indicates the mean ± SD of six brain slices. A summary of all experiments (*n* = 10) for each of the three peaks is provided in the lower panels. Connected data points derive from the same experiment and bars indicate means. Statistical analysis was performed using paired Student’s *t*-tests. (**B**) Hippocampal brain slices were processed like in (A) and stimulated by three consecutive strong electropulse series (500 pulses, 50 Hz; black arrows). The results of a representative experiment are shown in the upper panel. Each data point indicates the mean ± SD of six brain slices. A summary of all experiments (*n* = 10) for each of the three peaks is provided in the lower panels. Connected data points derive from the same experiment and bars indicate means. Statistical analysis was performed using paired Student’s *t*-tests. ^**^P < 0.01; *n.s.*, not significant.

To examine whether the neurotransmitter abnormalities in ether lipid-deficient mice manifest in compromised transmitter release, we loaded slices from hippocampus and parietal cortex of control and ether lipid-deficient mice with radiolabeled norepinephrine and evoked its release in superfusion chambers by different stimuli. In order to obtain the maximum amount of information from each experiment, we established a protocol involving two consecutive series of electropulses (100 pulses, 10 Hz, 40 mA, 0.5 ms) followed by a prolonged chemical, high KCl (40 mM KCl for 30 s) stimulus. Addition of the norepinephrine reuptake blocker desipramine between the two electrical stimuli increased the amount of norepinephrine released into the superfusate in both genotypes (Fig. 4A). Upon electrical stimulation, we did not detect any differences between the genotypes, neither with nor without addition of desipramine. However, the stronger chemical stimulation revealed a statistically highly significant decrease in [^3^H]-norepinephrine release from both hippocampal (30% reduction; Fig. 4A, third peak) and cortical slices (22% reduction; Supplementary Material, Fig. S5, third peak) upon ether lipid deficiency.

Because previous experiments using ether lipid-deficient synaptosomes had indicated changes in the Ca^2+^-dependent as well as Ca^2+^-independent portions of neurotransmitter release (25), we considered the possibility of altered Ca^2+^-independent release in our setting. However, shifting to Ca^2+^-free buffer virtually abolished neurotransmitter release in both WT and *Gnpat* KO brain slices (Supplementary Material, Fig. S6A), showing that release is essentially Ca^2+^-dependent under our experimental conditions.

Next, we assessed, whether either the type (chemical versus electrical) or the intensity (strong versus weak stimulation) of the stimulus caused the genotype effect in our release experiments. Thus, we modified the protocol and applied three consecutive series of strong electropulses (500 pulses, 50 Hz, 40 mA, 0.5 ms) allowing the release to return to baseline between pulse trains (Fig. 4B, upper panel). The initial pulse series induced a response in WT slices comparable in magnitude to that resulting from high KCl stimulation, whereas the two subsequent pulses evoked less norepinephrine release, suggesting a depletion of primed vesicles and/or [^3^H]-norepinephrine or an involvement of ion channel inactivation. Strikingly, the release from *Gnpat* KO slices was significantly decreased by all three stimuli compared to WT brain slices (Fig. 4B) reproducing our findings with high KCl stimulation. The genotype effect was the largest in the release elicited by the last pulse series, with a 28% reduction in ether lipid-deficient slices, compared to 21% in the first and 17% in the second pulse series. To further characterize the vesicular depletion in our model system, we exposed WT and *Gnpat* KO brain slices to a continuous (15 min) high-KCl stimulus (Supplementary Material, Fig. S6B). In WT cells, this provoked an initial peak followed by a continuous non-linear decrease of norepinephrine release. The area under the curve (AUC) was significantly reduced in *Gnpat* KO tissue (28%; Supplementary Material, Fig. S6B), mainly owing to a lower initial response with the later release well aligned between the two genotypes. Taken together, these results show that ether lipid-deficient synapses can respond adequately to low-intensity stimulation but are unable to tune up their response upon stimuli of higher intensity (either chemical or electrical).

### Levels of the norepinephrine transporter but not the serotonin transporter are affected in the ether lipid-deficient mouse brain

**Figure 5:**
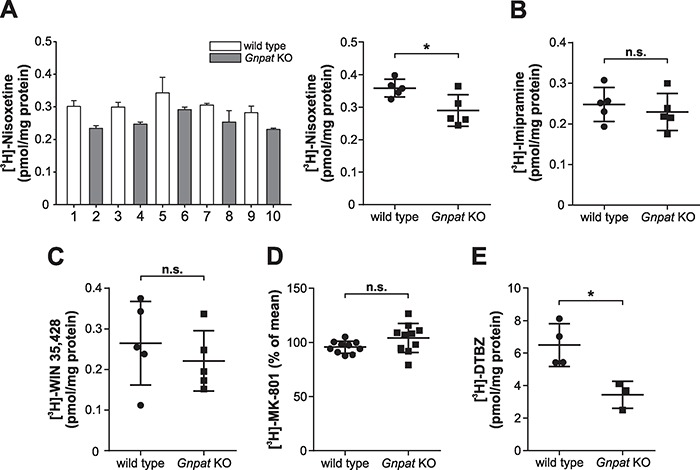
Binding assays targeting key proteins in synaptic transmission in cortical membrane and striatal vesicle preparations from WT and ether lipid-deficient mice. (**A**) Radioactive binding assays using the specific ligand [^3^H]-nisoxetine provided the levels of NET in cortical membrane fractions from WT and *Gnpat* KO mice (*n* = 5/genotype). The left graph shows the results of one representative experiment expressed as mean ± SD of technical triplicates. The numbers on the x-axis indicate individual mice. The right graph summarizes all results with individual data points representing means derived from two independent experiments. In addition, the group mean ± SD of all analyzed animals is depicted. (**B**) Radioactive binding assays using the specific ligand [^3^H]-imipramine provided the levels of SERT in cortical membrane fractions from WT and *Gnpat* KO mice (*n* = 5/genotype). Individual data (means of two independent experiments) and group mean ± SD of all analyzed animals are depicted. (**C**) Radioactive binding assays using the specific ligand [^3^H]-WIN 35,428 provided the levels of DAT in striatal membrane fractions from WT and *Gnpat* KO mice (*n* = 5/genotype). Individual data and group mean ± SD of all analyzed animals are depicted. (**D**) Radioactive binding assays using the specific ligand [^3^H]-MK-801 provided the levels of NMDA receptor in cortical membrane fractions from WT and *Gnpat* KO mice (*n* = 10/genotype). For technical reasons, all mice could not be analyzed in the same experiment; thus, data were normalized to the mean of all animals within each experiment. Individual data representing means of two independent experiments and group mean ± SD of all analyzed animals are depicted. (**E**) Radioactive binding assays using the specific ligand [^3^H]-DTBZ provided the levels of VMAT2 in striatal vesicular preparations from WT (*n* = 4) and *Gnpat* KO (*n* = 3) mice. Data are shown individually and as group mean ± SD. Statistical analysis for all panels was performed using two-tailed Student’s *t*-tests. ^*^P < 0.05; *n.s.*, not significant.

Alterations in the levels of several proteins involved in the neurotransmitter cycle, like the reuptake transporters at the presynaptic membrane (33,34), have been proposed to accompany changes in neurotransmitter levels or release. Therefore, we examined the levels of different markers, for which a compensatory regulation is conceivable. We first addressed the levels of the reuptake transporter for norepinephrine (norepinephrine transporter, NET) in crude cortical membrane fractions from WT and *Gnpat* KO mice by a binding assay using the specific ligand [^3^H]-nisoxetine. Here, we detected modestly, but statistically significantly, reduced binding in homogenates derived from *Gnpat* KO mice in comparison with WT homogenates (Fig. 5A). We extended our ligand binding studies to another reuptake transporter, the serotonin transporter (SERT) and, remarkably, did not find any differences in the binding of [^3^H]-imipramine, a drug preferably binding to SERT, but to a minor extent also to NET (Fig. 5B). Similarly, in binding assays using the dopamine transporter (DAT) ligand [^3^H]-WIN 35,428 in striatal crude membrane fractions, there were no alterations between WT and *Gnpat* KO homogenates (Fig. 5C).

The NMDA receptor, an abundant postsynaptic receptor for glutamate, is another prominent synaptic protein that could potentially be affected by ether lipid deficiency—either directly or via altered glutamate release (25)—but we observed no significant changes in binding of the specific ligand [^3^H]-MK-801 in cortical membrane fractions of ether lipid-deficient mice as compared to WT controls (Fig. 5D). Similar results were obtained with membrane preparations from the hippocampus, another brain region, in which glutamatergic synapses are abundant (data not shown). Besides providing information on potential compensatory changes, these data corroborate our findings from the western blot analyses (Fig. 3), indicating that the number of synapses is unaffected in ether lipid-deficient mice.

### The levels of the vesicular uptake transporter VMAT2 are reduced in the ether lipid-deficient striatum

To further explore the basis of the deficit in neurotransmitters, we turned our attention to vesicular uptake, which, in case of monoamines in the CNS, is accomplished by the vesicular monoamine transporter 2 (VMAT2). In order to assess whether VMAT2 is present at normal levels in ether lipid-deficient mice, we utilized the selective ligand dihydrotetrabenazine (DTBZ) in a radioactive binding assay. Intriguingly, DTBZ binding was reduced by almost 50% in striatal vesicular preparations derived from *Gnpat* KO mice compared to WT animals (Fig. 5E). These data indicate that a deficiency in VMAT2 may be limiting for the transport of neurotransmitters into synaptic vesicles and, hence, cause the depletion of monoamine neurotransmitters in ether lipid-deficient mice.

### Ether lipid-deficient mice show hyperactivity and stereotypy, restricted social interaction and deficient fear conditioning

**Figure 6:**
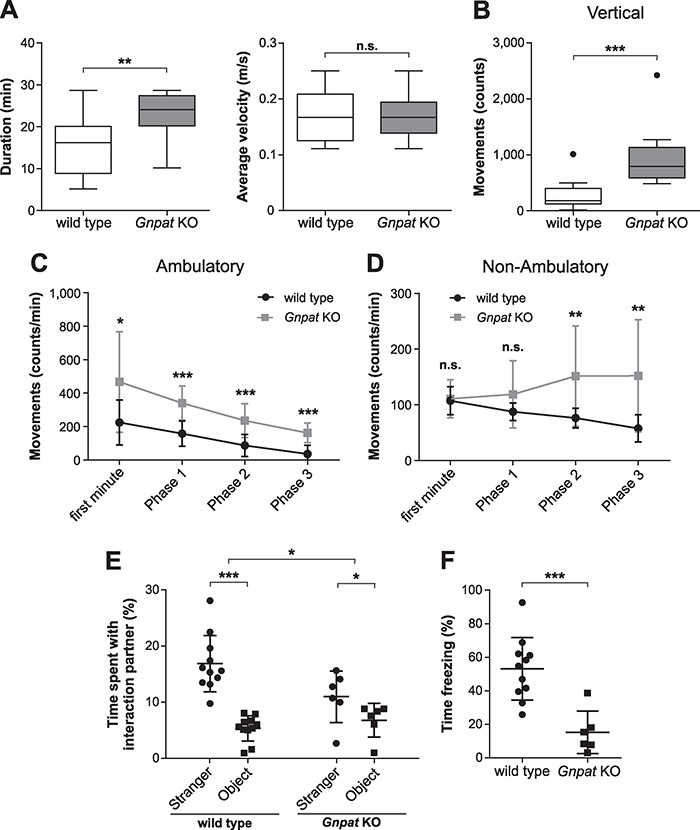
Ether lipid-deficient mice show hyperactivity and impaired sociability. (**A**) Results of WT (*n* = 13) and *Gnpat* KO (*n* = 11) mice in the closed-wheel test are shown as the time active during a 30-min period (left graph) and the average velocity while running (right graph). Box plots are drawn according to Tukey’s method, and statistical analysis was performed using two-tailed Student’s *t*-tests. The same cohort of mice was exposed to the open field paradigm for a total of 61 min, and vertical movements (**B**), ambulatory movements (**C**) and non-ambulatory movements (**D**) were recorded. Vertical movements were counted throughout the whole 61-min period, and box plots are drawn according to Tukey’s method. Statistical analysis was performed using Mann–Whitney *U*-test. For ambulatory and non-ambulatory movements, the first minute was analyzed separately and the remaining time was divided into three phases (phase 1: minute 2–21, phase 2: minute 22–41, phase 3: minute 42–61). Results are shown as mean ± SD, and statistical analysis was performed using two-tailed Student’s *t*-tests followed by Holm–Sidak correction for the repeated measurements. (**E**) Sociability was assessed by the three-chamber social interaction test, and the time spent interacting with an unfamiliar mouse (‘stranger’) or a neutral, novel object (‘object’) was quantified (WT: *n* = 11; *Gnpat* KO: *n* = 6). Results are shown as mean ± SD. Statistical analysis was performed using paired, two-tailed Student’s *t*-tests (for the comparison of the preference for stranger or object within each genotype) and repeated measures two-way ANOVA (for comparison of the genotypes; interaction effect: *F*(1,15) = 6.533; *P* = 0.022). (**F**) The contextual fear conditioning paradigm was applied to WT (*n* = 11) and *Gnpat* KO (*n* = 6) mice, and the duration of the freezing response is depicted as mean ± SD. Statistical analysis was performed using two-tailed Student’s *t*-test. ^***^P < 0.001; ^**^P < 0.01; ^*^P < 0.05; *n.s.*, not significant.

Deficits in neurotransmitter, particularly monoamine, levels or release have repeatedly been associated with hyperactivity (35,36). Ether lipid-deficient mice can be distinguished from their WT littermates by an increased level of general activity and abnormal behavioral patterns like stereotypic rearing in the home cage (own unpublished observation). Therefore, we applied several simple tests to obtain objective measures of the potential hyperactivity. First, we placed a running wheel in the home cage for 7 days allowing mice to use it at will (voluntary-wheel task). We observed a slight, statistically not significant trend toward increased running times of *Gnpat* KO mice compared to WT controls (Supplementary Material, Fig. S7). However, clear-cut results were obtained in the closed-wheel task, in which tested mice could not opt to leave the wheel, but could choose whether to rest or to run. Here, *Gnpat* KO mice spent significantly more time running than WT mice (Fig. 6A, left panel). They ran with similar speed as WT animals (Fig. 6A, right panel), which is remarkable given their motor impairment (26).

Next, the locomotor activity was determined in the open field test, a classical paradigm for assessment of hyperactivity. Over a trial time of 61 min, all vertical, ambulatory and non-ambulatory movements of the test animals were recorded separately. For the vertical movements, mainly reflecting rearing behavior, the cumulative counts were strikingly higher for *Gnpat* KO mice than for WT mice (Fig. 6B), confirming our previous observations in the home cage setting. For the analysis of ambulatory movements, reflecting normal forward motion, the first minute was separated from the remaining trial time (divided into three 20-min blocks), as it rather indicates exploratory behavior than locomotor activity. In both genotypes, we observed a continuous decrease throughout the entire trial, most likely due to decreasing interest in the new surroundings (Fig. 6C). However, at all time points examined, *Gnpat* KO mice showed significantly higher movement counts compared to WT mice. We identified a different pattern for non-ambulatory movements, which summarize non-forward movements like self-grooming and can be a measure of stereotypic behavior (31). No difference between the two genotypes could be detected in the first minute and in phase 1 of the trial (Fig. 6D). However, whereas the number of non-ambulatory movements decreased in the subsequent phases in WT animals, they increased in *Gnpat* KO animals probably replacing ambulatory movements, which declined at later stages of the trial. This reciprocal development in the time course of the paradigm led to statistically highly significant differences between WT and *Gnpat* KO animals in phases 2 and 3, in spite of substantial variation in this type of behavior, especially in the KO cohort. Overall, these findings corroborate the hyperactive phenotype of ether lipid-deficient mice.

To further investigate the behavioral phenotype of *Gnpat* KO mice, specifically in light of a potential relation between human neurodevelopmental disorders and ether lipid deficiency, we exposed them to the three-chamber social interaction task. In this experimental setting, test mice are provided the opportunity to explore two side chambers next to an empty entrance chamber: one with an unfamiliar mouse (‘stranger’), the other with a neutral, non-social object (i.e. a black Lego block). As anticipated, WT mice spent considerably more time interacting with the stranger mouse than with the neutral object (Fig. 6E). In contrast, ether lipid-deficient mice showed only a slight preference for the stranger over the object and the interaction time with the stranger was clearly reduced compared to WT littermates.

Conventional tests for cognition, employing for example the Morris water maze, cannot be reliably applied to *Gnpat* KO mice due to their visual impairments. We, thus, chose contextual fear conditioning as a paradigm to evaluate cognitive abilities of ether lipid-deficient mice. Remarkably, *Gnpat* KO mice showed a markedly reduced freezing response to the conditioning context manifesting in a statistically highly significant difference in freezing time between the genotypes (Fig. 6F). Summarized, ether lipid-deficient mice exhibit widespread alterations in specific aspects of behavior that may in part be triggered by impairments in neurotransmitter homeostasis and release.

## Discussion

Our studies revealed a consistent reduction by 20–40% in the levels of several neurotransmitters, particularly the monoamines and GABA, in cerebral homogenates of *Gnpat* KO mice. The levels of glutamate and glycine did not differ from controls, probably because alterations in the neurotransmitter fraction of these amino acids are masked by the pool for protein biosynthesis contributed by all cell types in the brain. Hence, we assume that neurotransmitter depletion is of general nature affecting all transmitter classes. Furthermore, in support of a ubiquitous neurotransmitter reduction throughout the nervous system, we recently identified lowered tissue levels of norepinephrine in cardiac homogenates of *Gnpat* KO mice (Dorninger *et al.*, manuscript in preparation).

Furthermore, our studies involving the dopaminergic nigrostriatal pathway indicate that the decrease in neurotransmitters is selective for synapses and does not affect neuronal cell bodies. Neurotransmitter deficits have been described in many late-onset neurological disorders but are in most of these, including Alzheimer’s disease (37), Parkinson’s disease (38) and Huntington’s disease (39), a direct consequence of neuronal degeneration and the resulting loss of synaptic structures. In contrast, neurodegeneration or degradation of synapses is not considered a general pathological feature of ether lipid deficiency but might be a factor in restricted brain areas (30) or very old ether lipid-deficient mice (24). However, our immunoblot analyses indicated no reduction in the presynaptic SNARE complex proteins synaptotagmin and synaptophysin in the cerebral cortex, where monoamine neurotransmitter levels were the most strikingly altered, in ether lipid deficiency, arguing against synaptic loss as cause for the neurotransmitter deficit. Similarly, radioligand binding assays revealed unchanged binding to the presynaptic reuptake transporters SERT and DAT in ether lipid-deficient samples, further underscoring that the number of synapses is normal. The small decrease in [^3^H]-nisoxetine binding in *Gnpat* KO samples can probably be explained by a compensatory downregulation of NET levels in response to the catecholamine neurotransmitter deficit. Such a feedback mechanism has been shown previously in rat brain, where the expression of NET is reduced upon depletion of norepinephrine (34).

A potential impact from the lack of plasmalogens on the vesicular uptake of neurotransmitters has been debated previously (21). Reduced adenosine triphosphate (ATP) levels and mitochondrial impairment, as discovered in synaptosomes of ether lipid-deficient mice (25), are bound to compromise the electrochemical gradient required for vesicular uptake, which is strictly ATP-dependent. Here, we found strikingly reduced levels of VMAT2 in striatal tissue homogenates of *Gnpat* KO mice. VMAT2, the only vesicular monoamine transporter in the nervous system, is expressed in all monoaminergic neurons and particularly abundant in the striatum on account of the numerous dopaminergic nerve terminals. Strongly reduced levels of VMAT2 in a mouse model (VMAT2 LO) are accompanied by decreased vesicular uptake and tissue levels of monoamines (40). Given that the cytosolic levels of monoamines are maintained, as an excess of cytosolic monoamines can produce neurotoxic metabolites and reactive species (41), reduced vesicular uptake likely leads to increased degradation of cytosolic neurotransmitters.

Several groups independently generated mice with a targeted deletion of the *Vmat2* gene. *Vmat2^−/−^* mice show almost complete depletion of monoamine neurotransmitters and die soon after birth (42–44), while *Vmat2^+/−^* mice, as the *Gnpat* KO mice in our study, display about 50% reduction of VMAT2 levels and are able to thrive. However, data on tissue monoamine levels of these heterozygous mice are conflicting, ranging from increased striatal dopamine levels (43), which appears puzzling in view of other literature, to considerably reduced levels of all monoamine neurotransmitters, in particular dopamine and serotonin, in whole-brain extracts of neonatal heterozygotes (42), the latter being similar to our findings in adult *Gnpat* KO mice. Interestingly, also in a *Drosophila* model of VMAT2 depletion, brain levels of monoamines decreased proportionally to the reduction of transporter levels (45).

Our studies mostly focused on monoaminergic neurons. However, we assume that other neurotransmitter systems are affected similarly by ether lipid deficiency, as indicated by the reduced levels of GABA in cerebral homogenates and, even more pronounced, in individual brain regions, like the cerebral cortex, of *Gnpat* KO mice. Cytosolic GABA concentrations are in equilibrium with the vesicular GABA pool (46). Consequently, it can be expected that GABA accumulating in the cytosol is efficiently removed by the degrading enzyme GABA transaminase, when vesicular uptake is impaired. In addition, the high similarity of vesicular uptake transporters for monoamines (VMAT) and GABA (vesicular GABA/inhibitory amino acid transporter, VGAT/VIAAT) implicates impaired uptake as a common cause for the depletion of the different neurotransmitters.

Our release experiments in hippocampal and cortical slices from *Gnpat* KO mice revealed impaired norepinephrine release upon strong electrical and chemical stimulation. These findings complement previous results from *Gnpat* KO synaptosomes showing reduced Ca^2+^-dependent release of glutamate and acetylcholine (25). It is tempting to speculate that the putatively impaired vesicular uptake and resulting reduction in neurotransmitter levels that we observed *in vivo* also led to reduced transmitter release in the *ex vivo* experiments. Here, though, vesicular uptake is presumably less limiting than *in vivo* due to the excess of radiolabeled neurotransmitter during loading and lack of replenishment in the superfusion chambers. Consequently, other molecular mechanisms have to be considered in this setting.

Synapses have a large capacity to compensate for abnormal neurotransmitter homeostasis. According to the current state of knowledge, synaptic vesicles are organized into different functional pools with the majority of vesicles residing in a so-called reserve (or resting) pool, which is not released under normal conditions (47,48). Theoretically, synapses could therefore adapt to deficits in neurotransmitter release by mobilizing increased numbers of vesicles from the reserve pool. However, it is debated whether these vesicles are released *in vivo* (48,49) or whether they serve other functions like the provision of proteins required for vesicle recycling (50).

Previous studies with *Gnpat* KO mice identified synaptosomal energy deficits and impaired respiration following depolarization with high KCl (25). These data bear striking similarity to our findings showing impaired neurotransmitter release upon strong depolarization in brain slices. Not only vesicular uptake but the entire synaptic vesicle cycle is strongly ATP-dependent, and healthy synapses adapt their level of ATP generation to synaptic activity (51). Conversely, synaptic ATP production is vital for proper synaptic function (51). It is plausible that in ether lipid deficiency, synaptic mitochondria cannot keep up with the increased energy demand upon strong stimulation, thus accounting for our findings. Considering the proposed role of plasmalogens in promoting membrane fusion and constriction (16,52), ether lipid deficiency most likely also directly affects the endo- and exocytotic processes of the synaptic vesicle cycle. Particularly, delayed recruitment and release of additional vesicles, especially under conditions of high demand, would explain our results in response to strong stimulation. Moreover, the concept that impaired vesicle fusion contributes to the observed reduction in norepinephrine release in ether lipid deficiency is strengthened by our recent studies of the neuromuscular junction in *Gnpat* KO mice, showing significant decreases in both the frequency of spontaneous vesicle fusion and the number of vesicles released upon stimulation (26).

Finally, our biochemical findings raised the question about potential physiological and phenotypic consequences of the presumed globally compromised neurotransmitter homeostasis and release. Notable aspects in this respect are (i) the behavioral phenotype of *Gnpat* KO mice, as described here (Fig. 6); (ii) the proposed genetic link between ether lipid deficiency and autism spectrum disorders in humans (53,54); and (iii) the presence of clinical features of autism or hyperactivity in patients with milder representations of RCDP (54,55). Together, these findings support the idea of an association between ether phospholipid deficits and neurodevelopmental disorders, which is substantiated by the observation of reduced plasmalogen levels in the plasma of autistic patients (56,57). Furthermore, patients suffering from autism spectrum disorders display deficits in the circulating levels of PUFAs (58). Plasmalogens are particularly rich in PUFAs, and our own results indicate decreased brain levels of docosahexaenoic acid in ether lipid deficiency (59). A role of PUFA insufficiency is also discussed in attention deficit hyperactivity disorder (ADHD) (60,61), probably the human disease best reflecting the hyperactive phenotype of *Gnpat* KO mice.

Considering that a variety of environmental and genetic factors have been implicated in the etiology of autism spectrum disorders (62,63) or ADHD (64), ether lipid deficiency may play a modulating rather than a causative role in the pathophysiology of these disorders. Dysregulated levels of neurotransmitters, particularly the monoamines, appear to be involved in both diseases (65,66), and mutations in SERT and DAT have been linked to autism and ADHD, respectively (67,68). Due to their wide range of impairments, *Gnpat* KO mice differ considerably from commonly used mouse models of autism or ADHD. However, stereotypic behavior like rearing or exaggerated self-grooming, a standard trait of mouse models of autism (69), is reliably observed in ether lipid-deficient mice. Furthermore, autism-like behavior in mice is often characterized by impaired sociability (70), as demonstrated here for *Gnpat* KO mice. On the other hand, increased activity in the home cage or the open field paradigm, particularly in later stages of the trial, is typical for animal models of ADHD (71). Certainly, the different behavioral properties cannot be viewed independently and must be discussed in the context of the complex phenotype of ether lipid-deficient mice. For example, the social interaction task may well be influenced by hyperactivity or, vice versa, stereotypic movements likely impact the counts in the open field task. Nevertheless, the combinatorial observation of features associated with both autism and ADHD in *Gnpat* KO mice, as first reported here, strengthens the concept of an association between ether lipid deficiency and behavioral traits related to human neurodevelopmental disorders.

An interesting model in this context is the so-called *coloboma* mouse (72), carrying a deletion affecting 20 genes, among them *Snap 25* encoding a component of the SNARE complex. Similar to the situation in *Gnpat* KO mice, the 50% reduction of SNAP-25 in the *coloboma* mouse presumably affects all synapses, independently of the neurotransmitter system. Most likely due to dysregulated levels and release of different transmitters (73,74), these mice show increased locomotor activity (75,76) and are widely considered as a model mimicking ADHD (77).

Altogether, various observations link neurodevelopmental disorders and the deficiency in ether lipids; our findings support this hypothesis and implicate a deficit in neurotransmitters as a common mechanistic feature. We provide novel insights into the physiological effects of ether lipid insufficiency and emphasize lipid alterations as a factor that warrants consideration in the development of neurodevelopmental disorders.

## Materials and Methods

### Mice

Mice with a targeted inactivation of the *Gnpat* gene (*Gnpat^tm1Just^,* MGI:2670462) have been described previously (23). The strain was maintained on an outbred C57BL/6 x CD1 background, and experimental cohorts with *Gnpat*^−/−^ (KO) and *Gnpat*^+/+^ (WT) littermates were obtained by mating heterozygous animals. Genotypes were determined at weaning by polymerase chain reaction as described previously (23) and confirmed at death. Mice had access to standard chow and water *ad libitum* and were housed in a temperature- and humidity-controlled room with 12:12-h light–dark cycle and a low level of acoustic background noise at the local animal facility of the Medical University of Vienna.

### Human tissue

Human de-identified *post-mortem* tissue from patients with ether lipid deficiency—either isolated (RCDP) or in the context of defective peroxisomal biogenesis (Zellweger spectrum disorders)—and controls was obtained from the NIH NeuroBioBank (University of Maryland, Baltimore, Maryland) and maintained in frozen state until analysis. Patient material was selected based on the availability of the brain regions of interest (caudate nucleus, primary motor cortex—Brodmann area 4). For every disease case, two matched control samples were selected according to the following criteria: age, gender, *post-mortem* interval and race. An overview of all cases used in this study is provided in Table S1. For one case (UMB_5391), Brodmann area 4 tissue was unavailable. Due to suspected contamination or inferior tissue quality, three tissue samples from the patient group (both brain regions of UMB_5391, cortex of UMB_4658) were excluded from analysis. For technical reasons, serotonin levels could not be determined in the cortex of one patient, norepinephrine could not be determined in the caudate nucleus of two controls and amino acids could not be determined in the caudate nucleus of one control.

### Determination of neurotransmitters

Mice were killed by cervical dislocation, their brains (cerebrum) isolated and immediately frozen on dry ice. Brain regions (parietal cortex, striatum and substantia nigra) were dissected in a frozen state. Particular care was taken to separate parietal cortex from striatal tissue. The analytical detection of neurotransmitters by using HPLC with electrochemical detection (monoamines) or fluorometric detection (amino acids) was performed as described in detail previously (31,78). Volumes for homogenization in 0.1 M perchloric acid containing 0.4 mM NaHSO_3_ were adapted to the region of interest (murine brain: whole cerebrum, 5 volumes; parietal cortex, 50 volumes; striatum and substantia nigra, 100 volumes; human brain tissues: 5 volumes). For normalization, 3,4-dihydroxybenzylamine (1–3 ng/100 μl) was used as internal standard. Purchased standards for all detected compounds were utilized for quantification.

### Preparation of crude cortical or striatal membrane fractions

Mice were sacrificed by cervical dislocation and brain regions dissected at 4°C. Tissue pieces were homogenized in 5 ml ice-cold Tris-Cl buffer (50 mM Tris, pH 7.4) or, for MK-801 binding, Tris-acetate buffer (50 mM Tris-acetate, 3 mM EDTA, pH 7.0) using a Teflon-glass tissue grinder (10 strokes). The volume was adjusted to 13 ml, and the samples were centrifuged at 36 000 × *g* (10 min, 4°C). The supernatant was discarded and the pellet resuspended in Tris-Cl or Tris-acetate buffer and centrifuged again. After resuspension, the homogenate was incubated at 37°C for 10 min. After another round of centrifugation, the sample was resuspended in Tris-Cl or Tris-acetate buffer (approximately 150 μl/mg tissue) and stored in aliquots at −80°C.

### Preparation of synaptic vesicles

The preparation of striatal synaptic vesicle fractions was performed using a series of differential centrifugation steps as described previously (79), and the obtained final pellet was stored at −80°C until further use.

### Radioactive binding assays

Binding assays were carried out based on previously published protocols (79–82). For nisoxetine, imipramine, WIN 35,428 and MK-801 binding assays, one aliquot of striatal or cortical membranes (corresponding to 7 mg of tissue) was thawed and transferred to a centrifuge tube rinsed with 0.04% Triton X-100. The volume was adjusted to 8 ml with incubation buffer (nisoxetine binding: 50 mM Tris-Cl, 300 mM NaCl, pH 7.4; imipramine binding: 50 mM Tris-Cl, 120 mM NaCl, 5 mM KCl, pH 7.4; WIN 35,428 binding: 50 mM Tris-Cl, 100 mM NaCl, pH 7.5; MK-801 binding: 10 mM Tris-acetate, pH 7.0), and the samples were centrifuged at 36 000 × *g* (Sorvall SM-24 rotor; 10 min, 4°C). The pellet was resuspended in 600 μl incubation buffer and the resulting homogenate used for binding assays. For each sample, triplicates were prepared and unspecific binding was determined in duplicates. Reaction mixes of 500 μl contained 100 μl of membrane suspension and radioactive ligands (WIN 35,428: American Radiolabeled Chemicals, St. Louis, Missouri; all others: PerkinElmer, Waltham, Massachusetts) at a final concentration of 2 nM ([^3^H]-nisoxetine, [^3^H]-imipramine), 1.4 nM ([^3^H]-WIN 35,428) or 5 nM ([^3^H]-MK-801) in incubation buffer. Unspecific binding was determined by using an excess of unlabeled, competitive ligands [nisoxetine binding: 10 μM desipramine (Tocris Bioscience, Bristol, UK); imipramine binding: 10 μM clomipramine (Tocris Bioscience, Bristol, UK); WIN 35,428 binding: 10 μM GBR 12909 (Tocris Bioscience, Bristol, UK); MK-801 binding: 60 μM S-ketamine (Sigma-Aldrich, St. Louis, Missouri)].

For DTBZ binding assays, pellets derived from vesicle preparations were resuspended in 200 μl sodium phosphate buffer (25 mM sodium phosphate, pH 7.7) and the resulting homogenate was used for binding assays. Reaction mixes of 100 μl contained 25 μl of vesicular suspension, 4 nM [2-^3^H]-DTBZ (American Radiolabeled Chemicals, St. Louis, Missouri). Unspecific binding was determined using an excess of unlabeled tetrabenazine (1 μM; American Radiolabeled Chemicals, St. Louis, Missouri).

After an incubation step (nisoxetine binding: 60 min at 25°C; imipramine binding: 30 min at 25°C; WIN 35,428 binding: 2 h at 0°C; MK-801 binding: 3 h at 23°C; DTBZ binding: 90 min at 30°C), bound radioligand was collected on glass fiber filters (pre-soaked in 0.3% polyethylenimine, except for DTBZ binding) using a Brandel harvester and the amount of radioactive label determined by scintillation counting. Protein levels were evaluated using the Bio-Rad (Hercules, California) Protein Assay Dye Reagent according to the manufacturer’s instructions.

### Western blot analysis

Immunoblotting was carried out as described previously (83). Primary antibodies used were rabbit anti-synaptotagmin I (Sigma-Aldrich, St. Louis, Missouri; cat.no. S 2177; 1:5000), rabbit anti-synaptophysin (Epitomics, Burlingame, California; cat.no. 1485–1; 1:2000), mouse anti-transferrin receptor (Invitrogen, Carlsbad, California; cat.no. 13–6800; 1:2000) and mouse anti-β-actin (Chemicon, Temecula, California; cat.no. MAB1501R; 1:40 000). Secondary antibodies were goat anti-mouse-HRP (Dako, Glostrup, Denmark; 1:20 000) and goat anti-rabbit-HRP (Bio-Rad, Hercules, California; 1:20 000). Quantitative analysis was done by using the rectangle tool of the Image Lab analysis software (version 5.2.1, Bio-Rad, Hercules, California).

### Neurotransmitter release from brain slices

Neurotransmitter release was evaluated as described previously (84) with minor modifications. WT and *Gnpat* KO mice were killed by cervical dislocation; the brain was removed, and the brain regions were dissected and placed in ice-cold low-calcium cell buffer (118 mM NaCl, 4.8 mM KCl, 0.2 mM CaCl_2_, 1.2 mM MgSO_4_, 25 mM NaHCO_3_, 1.2 mM K_2_HPO_4_, 0.03 mM Na_2_-EDTA, 11 mM glucose, 0.57 mM ascorbic acid, 0.5 mM fumaric acid, 5 mM sodium pyruvate; saturated with 95% O_2_/5% CO_2_). Hippocampus and cortex were cut into 300-μm slices using a McIlwain tissue chopper (Mickle Laboratory Engineering, Guildford, UK) and loaded in individual 40-μl droplets of loading buffer [low-calcium cell buffer +0.05 μM levo-[ring-2,5,6-^3^H]-norepinephrine (PerkinElmer, Waltham, Massachusetts), 0.5 μm clorgyline, 1 mM ascorbic acid] for 20 min at 37°C. The loaded slices were positioned in separate chambers of a superfusion device containing two platinum wire electrodes. A water bath ensured a constant temperature of 29°C. Residual radioactive label was removed by washing for 1 h with cell buffer (containing 2.5 mM CaCl_2_). The superfusion rate was 1 ml/min, and 2-min fractions were collected. Neurotransmitter release was triggered by electrical pulses (either 100 pulses, 10 Hz, 40 mA, 0.5 ms or 500 pulses, 50 Hz, 40 mA, 0.5 ms) generated by a stimulator (Hugo Sachs Elektronik, March, Germany) or chemically by application of 40 mM KCl (in cell buffer). In the standard protocol (Fig. 4A and Fig. S5), 0.5 μM desipramine (a reuptake inhibitor; Sigma-Aldrich, St. Louis, Missouri) was added after the first electropulse. In the strong electrostimulation protocol (Fig. 4B), cell buffer without clorgyline was used. At the end of the superfusion procedure, the slices were homogenized by sonication in a 1% SDS solution. Radioactivity in each fraction was determined by scintillation counting. Stimulation-evoked outflow was calculated as the difference between the total [^3^H] outflow during and after stimulation on the one hand, and the estimated basal outflow on the other hand, assuming that basal release follows a linear course. The released radioactivity was normalized to the total activity (sum of the released radioactivity and that remaining in the slice homogenates at the end of the experiment), and the peaks were analyzed using the AUC tool of the Prism software (GraphPad, version 6 for Windows).

### Behavioral analysis

All tests were conducted under conditions of dim light. Prior to all tests, mice were housed in individual cages for 2 weeks.

Voluntary wheel: A running wheel was placed in the test animal’s cage for 7 days, and the duration of voluntary exercise was tracked automatically using a commercial cyclometer (CM 211, Ciclosport, Gräfelfing, Germany).

Closed wheel: Mice were placed in a closed running wheel for 30 min, and the duration and running speed of exercise were tracked automatically.

Open field: Locomotor activity was evaluated as described previously (31,85) with slight modifications. Mice were placed individually in an activity meter (30 × 30 cm^2^; Opto Varimex, Columbus Instruments, Columbus, Ohio) and were allowed a few seconds for accommodation before starting the trial. Ambulatory, non-ambulatory and vertical movements were recorded automatically throughout the trial period of 61 min.

Social interaction: Experiments were conducted following a previously published protocol in a three-chambered sociability cage (Noldus Information Technologies, Wageningen, Netherlands) (86). Prior to each trial, the testing arena was cleaned with 70% ethanol and deionized water. For behavioral testing, the test mouse was placed in the central compartment and allowed to freely investigate the arena during a 30 min habituation period. On the following day, the mouse was again placed in the central compartment of the arena, with an unfamiliar age- and sex-matched mouse (‘stranger’) in a wired cage (allowing nose contact but no further physical contact) in one side chamber and a ‘dummy’ mouse (inanimate object, black Lego blocks) in the other. The location of the unfamiliar mouse and the object was alternated after each test animal. Trial time was 10 min. The unfamiliar ‘stranger’ mouse was trained to be held in the wire cage before testing and had no previous encounters with the subject mouse. For recording and analysis, the Ethovision12XT® program (Noldus Information Technologies, Wageningen, Netherlands) was used. To quantify the duration spent with either the stranger or the object, a specific area (‘sniff zone’) was drawn around the wire cages. The time the nose tip of the subject mouse was inside the sniff zone was used for calculations.

Contextual fear conditioning: Fear conditioning was conducted in behavior chambers (MED Associates, Fairfax, Vermont) located in sound-proof boxes according to a previously published procedure (87). Briefly, on two training days, mice were exposed to two consecutive electric foot shocks (0.6 mA, 2 s) in the behavior chamber. Contextual fear conditioning was tested 24 h after the last training day, by placing the mice in the same chamber for 5 min in the absence of the foot shock. The duration of the freezing response was used for automated, video-based analysis.

### Study approval and ethical considerations

Animal experiments were carried out in compliance with the 3Rs of animal welfare (replacement, reduction, refinement), and the number of animals was reduced to the estimated minimum necessary to obtain clear-cut, statistically significant results. The experiments were approved by the Institutional Animal Care and Use Committee of the Medical University of Vienna and the Austrian Federal Ministry of Science and Research (BMWF-5.011/0003-II/10b/2009 and BMWF-66.009/0010-II/3b/2014).

Experiments involving human tissue were performed in compliance with the principles stipulated in the Declaration of Helsinki, and the study plan was approved by the tissue access committee of the NIH NeuroBioBank prior to transfer of the tissue. Informed consent was obtained from all patients or legal representatives.

### Experimental design and statistical analysis

Prior to experiments, sample sizes were estimated using Java applets assuming a minimal statistical power of 0.85. To minimize variability, only adult male mice (littermates, as far as possible) were used at 3–8 months of age. Whenever possible, all biochemical and behavioral analyses were performed by an investigator blinded to the genotype of mice (in case of patient material, to disease condition). Details on the number of animals or replications for each experiment can be found in the Figure and Table Legends.

Routinely, the groups of WT (controls) and ether lipid-deficient mice (patients) were compared using two-tailed Student’s *t*-tests. Due to the apparent non-normal distribution of the variables, Mann–Whitney *U*-tests were applied in the analysis of monoamines in human patient material (Supplementary Material, Figs S3 and S4) as well as of vertical movement counts in the open-field test (Fig. 6B). Corrections for multiple comparisons were performed using the Bonferroni–Holm method, when analyzing control amino acids (Supplementary Material, Figs 1, S3 and S4); correction for repeated measurements was done using the Holm–Sidak method in the analysis of ambulatory and non-ambulatory movements of the open-field test (Fig. 6C and D). Social interaction (Fig. 6E) was analyzed using paired Student’s tests and repeated measures two-way analysis of variance (ANOVA). Statistical analysis was performed using Excel 2010 (Microsoft) and Prism (GraphPad, version 6 for Windows).

## Supplementary Material

Supplementary_Information_Dorninger_et_al_ddz040
