# Supplementary material for: Disturbed neurotransmitter homeostasis in ether lipid deficiency
Source: Hum Mol Genet. 2019 Feb 13;28(12):2046–61. doi: 10.1093/hmg/ddz040 (PMC6548223; doi:10.1093/hmg/ddz040)
Supplement: Supplementary_Information_Dorninger_et_al_ddz040 [file supplementary_information_dorninger_et_al_ddz040.pdf]

Supplementary Information

Disturbed Neurotransmitter Homeostasis in Ether Lipid Deficiency

Fabian Dorninger, Theresa König, Petra Scholze, Michael L. Berger, Gerhard Zeitler, Christoph Wiesinger, Anna Gundacker, Daniela D. Pollak, Sigismund Huck, Wilhelm W. Just, Sonja Forss-Petter, Christian Pifl, Johannes Berger

Supplementary Figure Legends

Figure S1: *The neurotransmitter deficits of Gnpat KO mice affect different brain regions.* The levels of the amino acids GABA (A) and taurine (B) as well as the monoamines norepinephrine (C) and serotonin (D) were determined in the parietal cortex (left column; WT: *n*=12, *Gnpat* KO: *n*=11), the striatum (middle column; *n*=7/genotype) and the substantia nigra (right column; WT: *n*=12, *Gnpat* KO: *n*=11) by using HPLC. Bars represent group means ± SD and statistical analysis was performed using two-tailed Student's t-tests. ***$P < 0.001$; *$P < 0.05$; *n.s.*, not significant. The displayed serotonin data were used for the calculation of the neurotransmitter/metabolite ratios presented in Table 1.

Figure S2: *Uncropped western blots for the data presented in Figure 3.* Numbers indicate the apparent MWs (in kDa) of a commercial, pre-stained protein marker run next to the samples. Bands corresponding to the expected MWs of synaptotagmin (65 kDa), actin (43 kDa), synaptophysin (34 kDa) and transferrin receptor (95 kDa) are indicated by arrows.

Figure S3: *No major changes in neurotransmitter levels in the parietal cortex of human patients with ether lipid deficiency.* The levels of dopamine (A), norepinephrine (B), serotonin (C), GABA (D), glycine (E), glutamate (F) and taurine (G) were determined in homogenates of parietal cortex (Brodmann area 4) tissue from controls (*n*=23) and ether lipid (EL)-deficient patients (*n*=7 for panel C, *n*=8 for all other panels) by using HPLC. Statistical analysis was performed using Mann-Whitney U-tests (panels (A)-(C)) and two-tailed Student's t-tests (panels (D)-(G)). Graphs depict individual data together with group means (horizontal line). The patient group involves one RCDP case (red-colored values) and 7 cases diagnosed with Zellweger spectrum disorders (see Table S1). (H) As a control, the levels of several proteinogenic amino acids were analyzed. Bars represent group means ± SD. Statistical analysis was performed using two-tailed Student's t-tests followed by Bonferroni-Holm correction for multiple comparisons. **$P < 0.01$; *n.s.*, not significant. The displayed dopamine and serotonin data were used for the calculation of the neurotransmitter/metabolite ratios presented in Table S2.

Figure S4: *No major changes in neurotransmitter levels in the caudate nucleus of human patients with ether lipid deficiency.* The levels of dopamine (A), norepinephrine (B), serotonin (C), GABA (D), glycine (E), glutamate (F) and taurine (G) were determined in homogenates of caudate nucleus tissue from controls (*n*=24 for panels A and C, *n*=22 for panel B and *n*=23 for panels E-H) and ether lipid (EL)-deficient patients (*n*=9) by using HPLC. Statistical analysis was performed using Mann-Whitney U-tests (panels (A)-(C)) and two-tailed Student's t-tests (panels (D)-(G)). Graphs depict individual data together with group means (horizontal line). The patient group involves one RCDP case (red-colored values) and 8 cases diagnosed with Zellweger spectrum disorders (see Table S1). (H) As a control, the levels of several proteinogenic amino acids were analyzed. Bars represent group means ± SD. Statistical analysis was performed using two-tailed Student's t-tests followed by Bonferroni-Holm correction for multiple comparisons. *$P < 0.05$; *n.s.*, not significant. The displayed dopamine and serotonin data were used for the calculation of the neurotransmitter/metabolite ratios presented in Table S2.

Figure S5: *Norepinephrine release from cortical slices mimics findings obtained in the hippocampus*. By analogy with hippocampal slices (cf. Fig. 4), parietal cortex slices were loaded with [^{3}H]-norepinephrine and neurotransmitter release was induced by two consecutive electropulse series (100 pulses, 10Hz; first two peaks; black arrows) and exposure to 40 mM KCl for 30 s (third peak; green arrow). Desipramine was added after the first electrical stimulus. The results of a representative experiment are shown in the upper panel. Each data point indicates the mean ± SD of 6 brain slices. A summary of all experiments (*n*=10) for each of the three peaks is provided in the lower panels. Connected data points derive from the same experiment and bars indicate means. Statistical analysis was performed using paired Student's t-tests. **$P < 0.01$; *n.s.*, not significant

Figure S6: *Release experiments in cortical slices reveal no difference in Ca^{2+}-independent release and confirm the impairment upon strong stimulation in ether lipid-deficient mice.* (A) Slices were loaded with [^{3}H]-norepinephrine and neurotransmitter release was induced by two consecutive electropulse series (100 pulses, 10Hz; first two peaks; black arrows) and exposure to

40 mM KCl for 30 s (third peak; green arrow). After the first electropulse, the superfusion buffer was deprived of Ca^{2+}. The results of a representative experiment are shown in the upper graph. Each data point indicates the mean ± SD of 6 brain slices. A summary of all experiments (*n*=6) for peaks 1 and 3 is provided in the lower graphs. Connected data points derive from the same experiment and bars indicate means. Statistical analysis was performed using paired Student's t-tests. A quantitative analysis of the second peak was omitted, as it was indistinguishable from the baseline in several experiments. (B) Neurotransmitter release was induced by exposure to 40 mM KCl, which was maintained throughout the experiment. The results of a representative experiment are shown in the upper panel. Each data point indicates the mean ± SD of 6 brain slices. A summary of all experiments (*n*=3) is provided in the lower graph. Connected data points derive from the same experiment and bars indicate means. Statistical analysis was performed using a paired Student's t-test. $^{**}P < 0.01$; *n.s.*, not significant

Figure S7: *Voluntary-wheel testing does not show altered voluntary running behavior of ether lipid-deficient mice.* Results of the voluntary-wheel test are shown as the time that WT (*n*=13) and *Gnpat* KO (*n*=11) mice spent running in an open wheel during a 7-day-period. The box plot is drawn according to Tukey's method and statistical analysis was performed using a two-tailed Student's t-test. *n.s.*, not significant

Figure S1

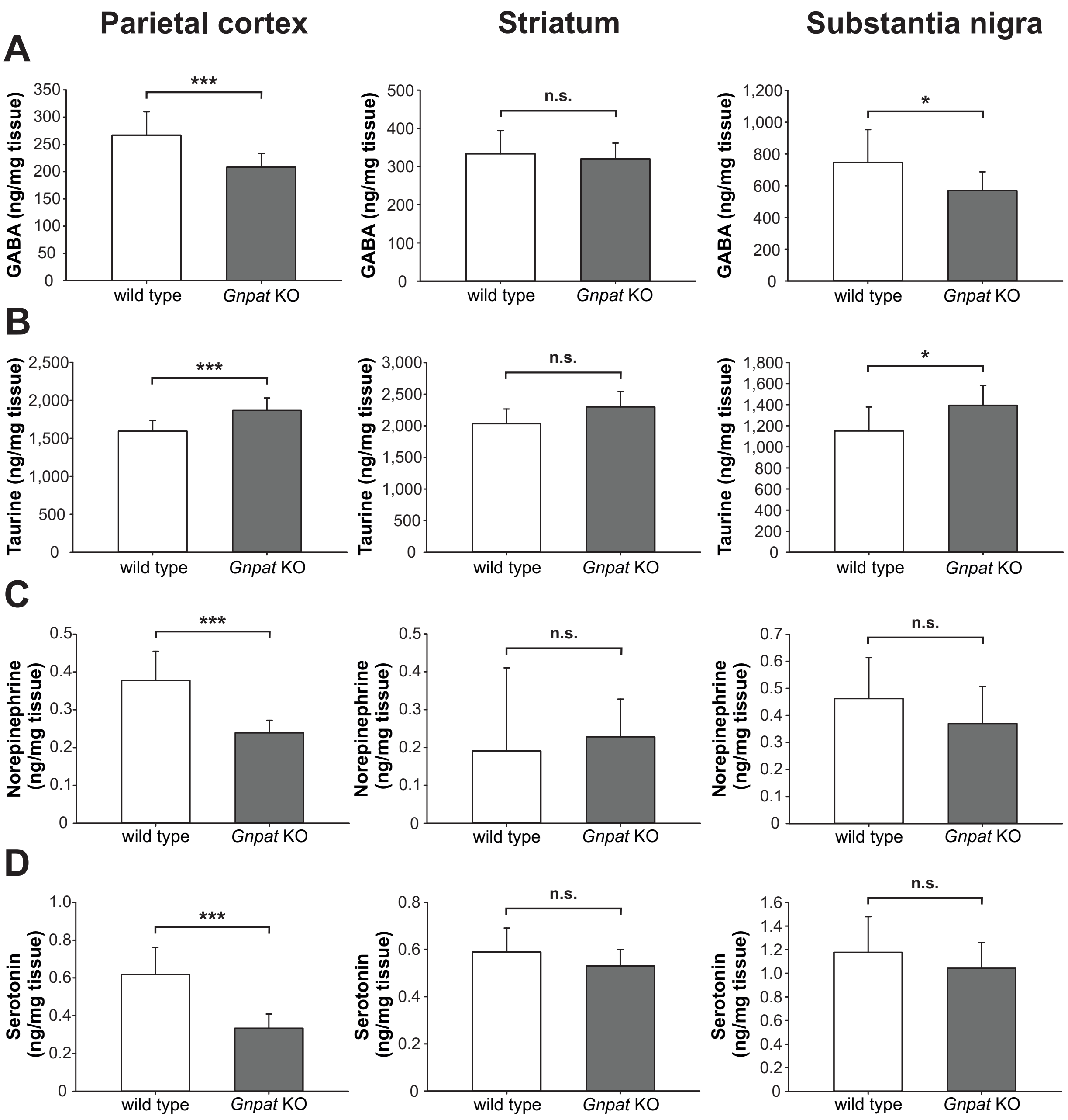

Parietal cortex
Striatum
Substantia nigra
A
B
C
D
GABA (ng/mg tissue)
Taurine (ng/mg tissue)
Norepinephrine (ng/mg tissue)
Serotonin (ng/mg tissue)
***
n.s.
*
wild type
Gnpat KO

Figure S2

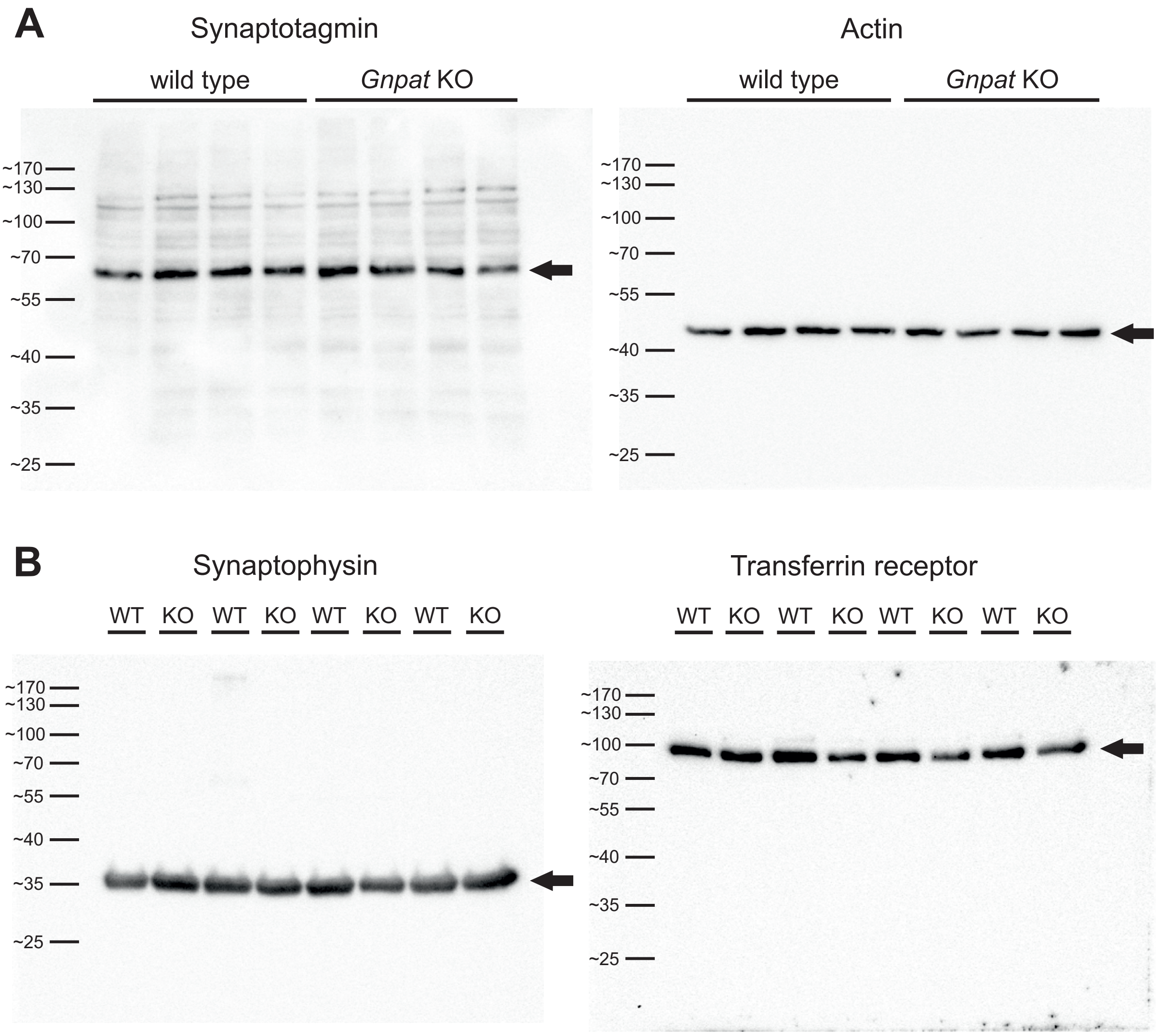
A
Synaptotagmin
wild type
Gnpat KO
~170
~130
~100
~70
~55
~40
~35
~25
Actin
wild type
Gnpat KO
~170
~130
~100
~70
~55
~40
~35
~25
B
Synaptophysin
WT KO WT KO WT KO WT KO
~170
~130
~100
~70
~55
~40
~35
~25
Transferrin receptor
WT KO WT KO WT KO WT KO
~170
~130
~100
~70
~55
~40
~35
~25

Figure S3

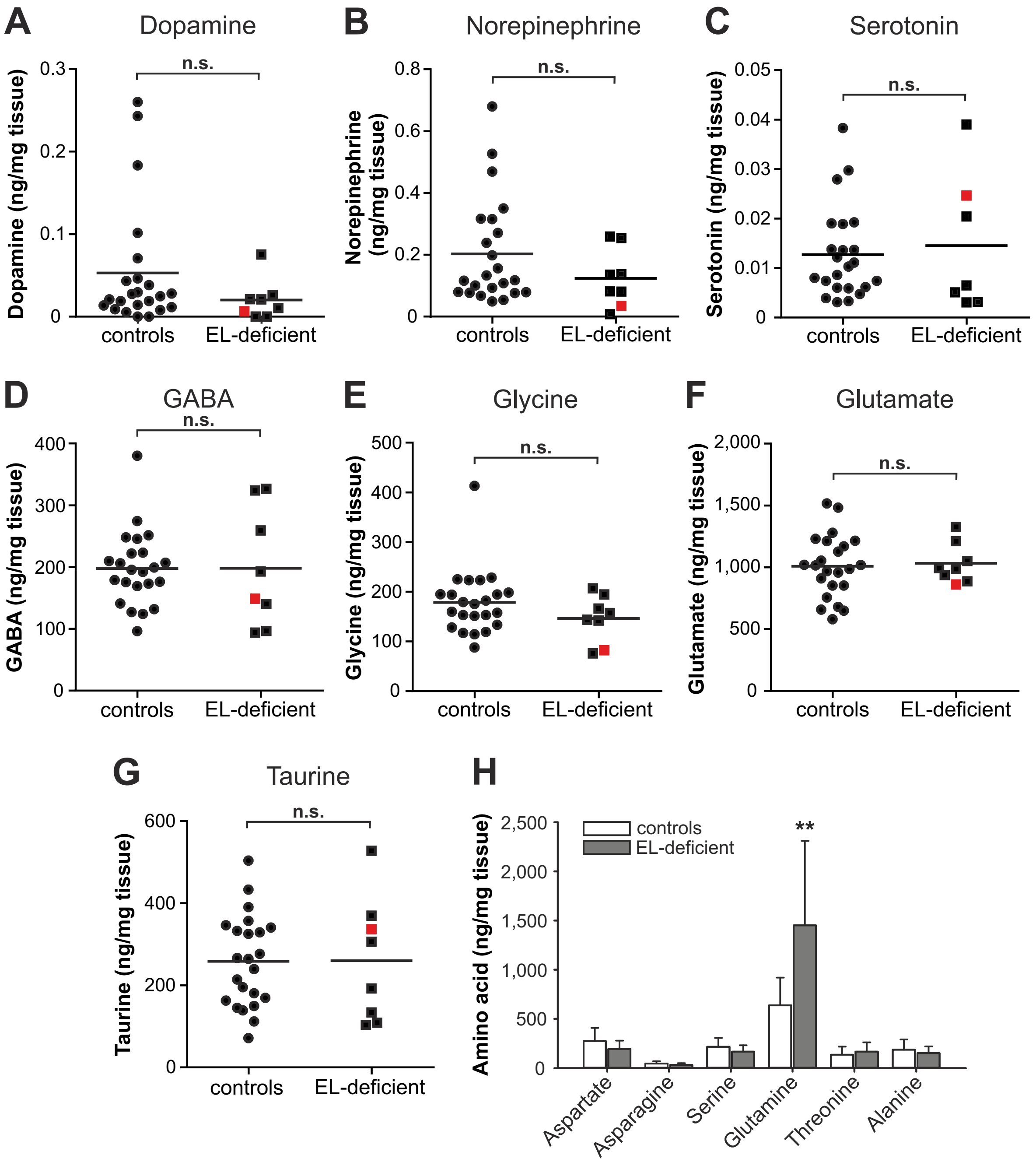
A
Dopamine
Dopamine (ng/mg tissue)
0.3
0.2
0.1
0
n.s.
controls
EL-deficient
B
Norepinephrine
Norepinephrine (ng/mg tissue)
0.8
0.6
0.4
0.2
0
n.s.
controls
EL-deficient
C
Serotonin
Serotonin (ng/mg tissue)
0.05
0.04
0.03
0.02
0.01
0
n.s.
controls
EL-deficient
D
GABA
GABA (ng/mg tissue)
400
300
200
100
0
n.s.
controls
EL-deficient
E
Glycine
Glycine (ng/mg tissue)
500
400
300
200
100
0
n.s.
controls
EL-deficient
F
Glutamate
Glutamate (ng/mg tissue)
2,000
1,500
1,000
500
0
n.s.
controls
EL-deficient
G
Taurine
Taurine (ng/mg tissue)
600
400
200
0
n.s.
controls
EL-deficient
H
Amino acid (ng/mg tissue)
2,500
2,000
1,500
1,000
500
0
controls
EL-deficient
**
Aspartate
Asparagine
Serine
Glutamine
Threonine
Alanine

Figure S4

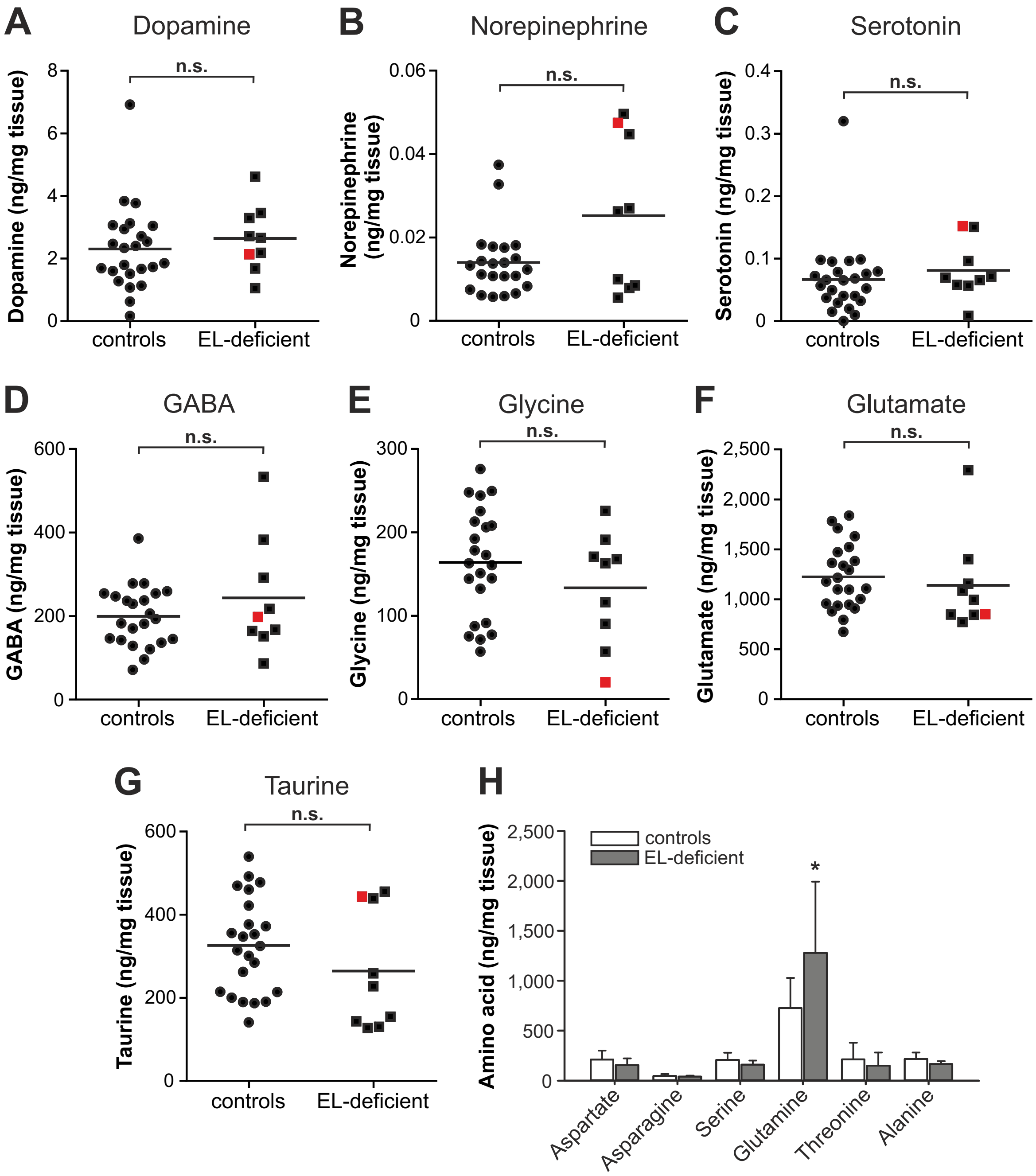
A
Dopamine
Dopamine (ng/mg tissue)
n.s.
controls
EL-deficient
B
Norepinephrine
Norepinephrine (ng/mg tissue)
n.s.
C
Serotonin
Serotonin (ng/mg tissue)
n.s.
D
GABA
GABA (ng/mg tissue)
n.s.
E
Glycine
Glycine (ng/mg tissue)
n.s.
F
Glutamate
Glutamate (ng/mg tissue)
n.s.
G
Taurine
Taurine (ng/mg tissue)
n.s.
H
Amino acid (ng/mg tissue)
controls
EL-deficient
*
Aspartate
Asparagine
Serine
Glutamine
Threonine
Alanine

Figure S5

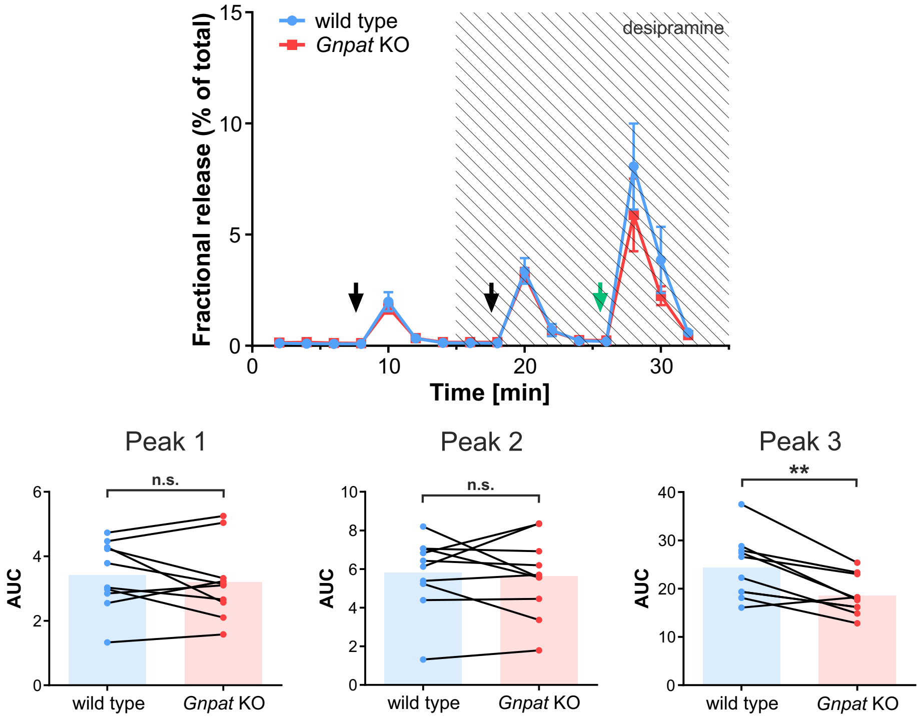

wild type
Gnpat KO
desipramine
Fractional release (% of total)
Time [min]
Peak 1
Peak 2
Peak 3
n.s.
n.s.
**
AUC
wild type
Gnpat KO

Figure S6

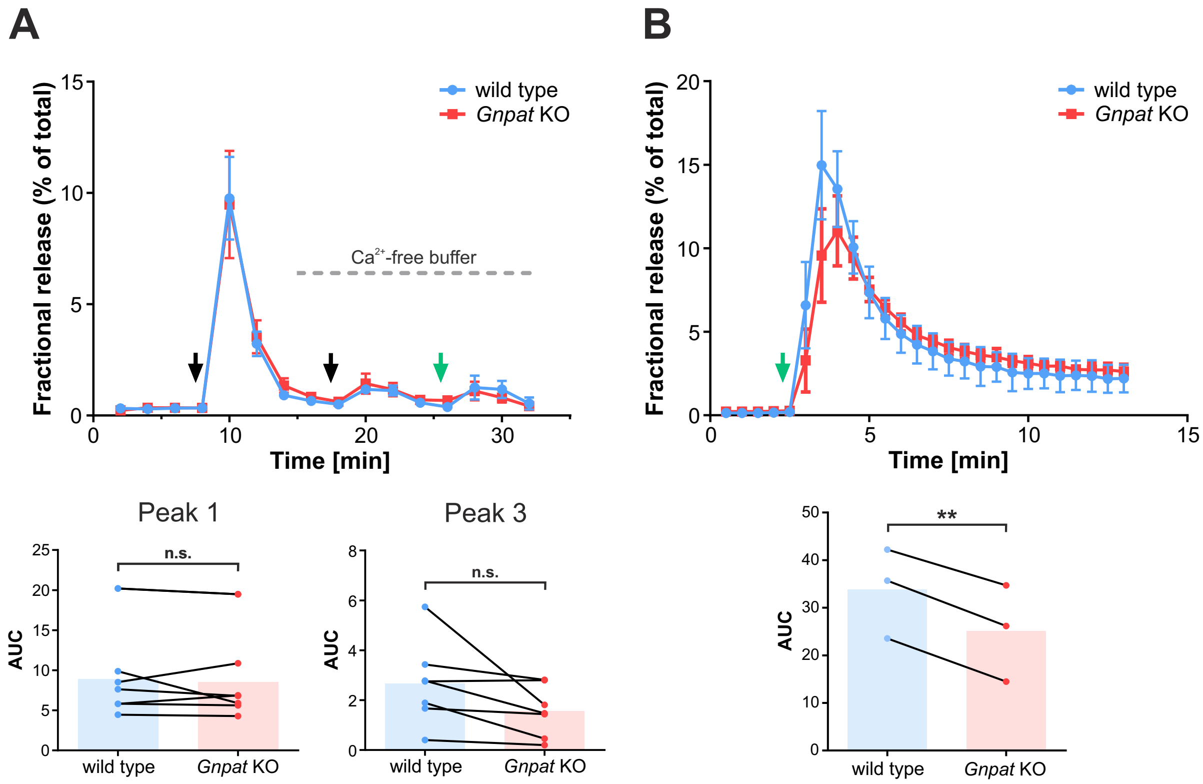

A
B
Fractional release (% of total)
Time [min]
wild type
Gnpat KO
Ca²⁺-free buffer
Peak 1
Peak 3
AUC
n.s.
**
wild type
Gnpat KO

Figure S7

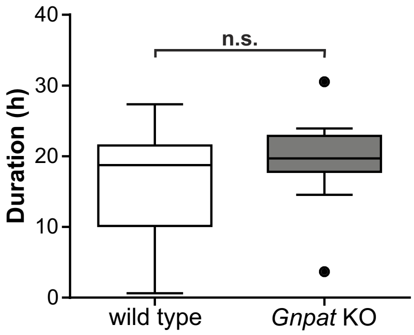

Table S1: Overview of human cases of ether lipid deficiency and controls

Diagnosis	ID	Age (years/days)	Sex	Race	Post-mortem interval (h)	Cause of death
**RCDP**	M3777M	0/181	m	Caucasian	3	disorder
**Zellweger Syndrome**	UMB_0150	0/63	m	Caucasian	22	disorder
**Zellweger-related (neonatal ALD)**	UMB_0765	34/234	f	Caucasian	7	multi-organ failure
**Zellweger Syndrome**	UMB_1202	0/181	m	American Indian	4	disorder
**Zellweger Syndrome**	UMB_1425	1/154	m	Caucasian	2	disorder
**Zellweger spectrum (neonatal ALD, PEX1 defect)**	UMB_4658	18/15	m	Caucasian	18	adrenoleuko-dystrophy, complications
**Zellweger spectrum (neonatal ALD, PEX1 defect)**	UMB_4753	34/37	m	Caucasian	28	cardiac arrest
**Zellweger spectrum (neonatal ALD)**	UMB_4766	5/139	m	Caucasian	10	disorder
**Zellweger Syndrome**	UMB_5096	0/45	m	Caucasian	12	disorder
**Zellweger Syndrome**	UMB_5861	6/262	f	Caucasian	27	disorder
**SIDS/Control**	M3440M	0/54	m	Caucasian	24	SIDS
**Control**	UMB_1055	0/96	m	Caucasian	12	broncho-pneumonia
**Control**	UMB_1210	0/68	m	African American	25	asphyxia
**Control**	UMB_1377	5/343	f	Caucasian	20	drowning
**Control**	UMB_1488	1/137	m	African American	21	head and neck injuries
**Control**	UMB_1609	33/17	f	Caucasian	24	coronary artery thrombosis
**Control**	UMB_1904	0/100	m	African American	14	dehydration
**Control**	UMB_4327	5/266	f	African American	24	respiratory failure
**Control**	UMB_4332	5/241	m	African American	18	pseudomonas broncho-pneumonia
**Control**	UMB_4353	0/34	m	African American	5	probable overlay
**Control**	UMB_4374	0/84	f	Caucasian	44	SIDS
**Control**	UMB_4376	0/200	m	Caucasian	25	probable asphyxation
**Control**	UMB_4391	0/27	m	Caucasian	13	asphyxia by suffocation
**Control**	UMB_4593	33/24	m	Caucasian	8	cardiac arrhythmia
**Control**	UMB_4848	16/271	m	Caucasian	15	accidental drowning
**Control**	UMB_4901	0/61	f	African American	28	asphyxia due to overlay
**Control**	UMB_4916	19/47	m	Caucasian	5	accidental drowning
**Control**	UMB_5180	1/262	m	Caucasian	25	drowning
**Control**	UMB_5391	8/286	m	Caucasian	12	drowning
**Control**	UMB_5408	6/309	m	African American	16	drowning
**Control**	UMB_5558	5/345	m	Caucasian	19	anomalous left coronary artery (complications)
**Control**	UMB_5622	34/254	m	Caucasian	11	complications of obesity
**Control**	UMB_5759	34/127	m	Caucasian	28	atherosclerotic cardiovascular disease
**Control**	UMB_5947	0/179	m	Caucasian	11	asphyxia

Abbreviations: ALD, adrenoleukodystrophy; PEX, peroxin; RCDP, rhizomelic chondrodysplasia punctata; SIDS, sudden infant death syndrome

Table S2: Levels of monoamine neurotransmitter metabolites and metabolite-to-neurotransmitter ratios in caudate nucleus and parietal cortex (Brodmann area 4) of controls and ether lipid-deficient patients

Metabolite (brain region)	Controls[a]	Ether lipid-deficient patients[b]
Serotonin metabolites		
5-HIAA (PC) (ng/mg tissue)	0.047 ± 0.031	0.141 ± 0.108***
5-HIAA/5-HT[c] (PC) (molar ratio)	4.137 ± 3.056	15.160 ± 10.178***
5-HIAA (CN) (ng/mg tissue)	0.192 ± 0.136	0.387 ± 0.386*
5-HIAA/5-HT[c] (CN) (molar ratio)	3.787 ± 3.485	4.394 ± 2.467
Dopamine metabolites		
DOPAC (PC) (ng/mg tissue)	0.022 ± 0.029	0.105 ± 0.186*
DOPAC/DA[c] (PC) (molar ratio)	0.532 ± 0.508	0.949 ± 1.639
HVA (PC) (ng/mg tissue)	0.156 ± 0.148	0.147 ± 0.122
HVA/DA[c] (PC) (molar ratio)	4.717 ± 3.730	9.700 ± 11.377
DOPAC (CN) (ng/mg tissue)	0.534 ± 0.408	0.452 ± 0.673
DOPAC/DA[c] (CN) (molar ratio)	0.536 ± 1.550	0.158 ± 0.231
HVA (CN) (ng/mg tissue)	2.667 ± 0.790	3.252 ± 1.546
HVA/DA[c] (CN) (molar ratio)	1.591 ± 2.208	1.099 ± 0.449

Abbreviations: CN, caudate nucleus; DA, dopamine; DOPAC, 3,4-dihydroxyphenylacetic acid; 5-HIAA, 5-hydroxyindoleacetic acid; 5-HT, serotonin; HVA, homovanillic acid; PC, parietal cortex

[a] PC: *n*=23 (no DOPAC/DA and HVA/DA ratios could be calculated for two cases owing to undetectable DA levels); CN: *n*=24 (no HIAA/5-HT ratio could be calculated for one case owing to undetectable 5-HT levels)

[b] PC: *n*=8 (1 RCDP, 7 Zellweger spectrum; no HIAA/5-HT ratio could be calculated for one case due to technical issues in 5-HT determination; no DOPAC/DA and HVA/DA ratios could be calculated for two cases owing to undetectable DA levels); CN: *n*=9 (1 RCDP, 8 Zellweger spectrum)

[c] Dopamine and serotonin values are derived from the data sets displayed in Supp. Figs. 4 (PC) and 5 (CN).

All values are means ± SD. ****P* < 0.001, **P* < 0.05 (two-tailed Student's t-test)
